# Investigating the effects of antipsychotics on brain insulin action: Study protocol for a multi-modality magnetic resonance imaging (MRI) study in healthy controls

**DOI:** 10.1371/journal.pone.0277211

**Published:** 2022-11-28

**Authors:** Nicolette Stogios, Laurie Hamel, Emily Smith, Marcos Sanches, Gary Remington, Aristotle Voineskos, Satya Dash, Ariel Graff-Guerrero, Margaret Hahn, Sri Mahavir Agarwal

**Affiliations:** 1 Institute of Medical Science, University of Toronto, Toronto, Ontario, Canada; 2 Centre for Addiction and Mental Health (CAMH), Toronto, Ontario, Canada; 3 Krembil Centre for Neuroinformatics, Centre for Addiction and Mental Health (CAMH), Toronto, Ontario, Canada; 4 Department of Psychiatry, University of Toronto, Toronto, Ontario, Canada; 5 Toronto General Hospital Research Institute, University Health Network (UHN), Toronto, Ontario, Canada; 6 Banting and Best Diabetes Centre (BBDC), University of Toronto, Toronto, Ontario, Canada; PLoS ONE, UNITED STATES

## Abstract

Antipsychotics (APs) are the cornerstone of treatment for schizophrenia (SCZ) but are unfortunately associated with serious metabolic adverse effects including weight gain and type 2 diabetes. The pathophysiology of AP-induced metabolic dysfunction is largely undetermined. Brain insulin resistance has been posited to be at the cross-roads of many cognitive and metabolic disorders, and disruption of central insulin action has emerged as a possible explanatory mechanism underlying AP induced metabolic dysfunction. Previous studies suggest that change in neuroimaging-based parameters with intranasal insulin administration can be leveraged to investigate brain insulin resistance. In this proof-of-concept study, we will utilize neural signatures of insulin action in the brain to examine if APs disrupt brain insulin signaling. It is hypothesized that: 1) intranasal insulin (INI), but not intranasal placebo (INP), will change cerebral blood flow and resting state connectivity, as well as increase glutamate levels in the striatum and dorsolateral prefrontal cortex; 2) oral olanzapine (OLA), but not oral placebo (PL), will inhibit the effect of INI on these parameters. Thirty-two healthy volunteers will undergo a single blind, cross-over design, wherein all participants receive the following four treatment combinations, 2–6 weeks apart, in a random sequence: INP + PL, INP + OLA, INI + PL, and INI + OLA. Participants will undergo an MRI-based assay of brain insulin resistance 15 minutes after administering 160 IU INI or INP. The scanning protocol includes resting and task-based functional MRI, arterial spin labelling, and proton magnetic resonance spectroscopy. Demonstrating that OLA can acutely induce brain insulin resistance is clinically relevant to metabolic health in SCZ. Evidence of brain insulin resistance induced by acute AP dosing can inform the early use of adjunctive insulin sensitizers for the treatment of metabolic comorbidities associated with AP treatment in severe mental illness.

Trial registration

**ClinicalTrials.gov Registration**: NCT03741478.

## Introduction

Antipsychotic (AP) medications are the cornerstone of treatment for schizophrenia (SCZ) spectrum disorders [1, 2], with increasing off-label use in children and adolescents [3]. Unfortunately, APs, and second-generation APs (SGA) in particular, are associated with serious metabolic adverse effects including significant weight gain, impaired glucose control, and dyslipidemia [4–7]. Such metabolic disturbances occur rapidly after exposure to APs [8, 9], with olanzapine and clozapine representing two SGAs with the greatest metabolic liability.

Recently, brain insulin resistance has been posited to lie at the cross-roads of many cognitive and metabolic disorders [10], and disruption of central insulin action has emerged as a plausible contributor to AP-induced metabolic dysfunction. The effect of APs on central insulin sensing is a novel area of investigation as the brain was long considered an insulin-insensitive organ [10]. However, it is now well-established that insulin within the central nervous system (CNS) has modulating effects on processes such as appetite regulation, metabolism, behaviour, and cognition [10]. For instance, using the pancreatic euglycemic clamp technique to separate central and peripheral insulin effects, it has been established that an intracerebroventricular (ICV) infusion of insulin in rodents, and intranasal administration of insulin in humans, results in a decrease in endogenous glucose production by the liver [11–13]. Interestingly, using this same gold-standard technique, our group has shown that an acute dose of olanzapine (OLA) can completely abolish the ability of a central insulin stimulus to decrease hepatic glucose production in rats [14]. This suggests that APs have a direct anti-insulin action in the brain that is independent of weight gain. Endogenous glucose production is a major source of fasting and post-absorptive hyperglycemia in type 2 diabetes; therefore, disruption of the central insulin regulation of hepatic glucose production poses patients on APs at a greater risk for developing glucose dysregulation.

Furthermore, the effect of central insulin on brain functioning and regulating cognition has also been demonstrated. Studies employing neuroimaging techniques with intranasal insulin (INI) administration have shown that insulin enhances connectivity in the default-mode network (DMN) between prefrontal brain regions and the hippocampus [15], reduces cerebral blood flow in the hypothalamus and prefrontal cortex (PFC) [16], and increases perfusion in the insular cortex [17]. Infusion of high concentrations of peripheral insulin also increases glutamate levels in the frontal and temporal cortex [18], but only in non-insulin resistant individuals. Interestingly, insulin resistance has specifically been correlated with lower striatal glutamate [19]. Moreover, studies in healthy, normal weight humans have revealed that INI administration improves declarative, working and verbal memory, as well as visuospatial function [10, 20]; improvements in visuospatial and verbal fluency performance have also been related to changes in regional vasodilation [16, 17]. Notably, these cognitive domains are disrupted in AP-treated patients with SCZ. Additionally, APs are known to normalize concentrations of glutamate within the striatum [21], which is otherwise high in unmedicated first episode SCZ patients [22]. Thus, with evidence to suggest an anti-insulin effect of APs, it is possible that APs might interact with cognitive processes through interruption of insulin-mediated processes on brain activity, perfusion and neurotransmission in salient brain regions [23, 24].

Taking the above findings together, INI-induced changes in the brain can be exploited and utilized as an “assay” of insulin activity, where absence of these changes can be interpreted as a sign of brain insulin resistance. Indeed, perfusion changes have been found to be absent or attenuated in participants with insulin resistance or high visceral fat [18, 25], demonstrating that neuroimaging-based parameters can be used to investigate the presence of insulin resistance in the brain. Thus, in this proof-of-concept study, we will utilize well-characterized neural signatures of brain insulin action to examine if APs disrupt brain insulin signaling in humans. We have chosen to 1) use olanzapine as the representative AP given its high metabolic liability, 2) study healthy volunteers to avoid confounding effects of psychiatric illness on glucose metabolism and cognition, and 3) utilize acute AP dosing to avoid adiposity changes, allowing us to dissect whether APs directly inhibit brain insulin action in humans.

## Objectives & hypotheses

### Objectives

The two objectives of this study are: 1) To examine if intranasal insulin (INI) induces any changes in cerebral blood flow, resting state connectivity, glutamate levels within the striatum and dorsolateral prefrontal cortex (DLPFC), and visuospatial task performance, as compared to intranasal placebo (INP); 2) To examine if olanzapine (OLA) acutely inhibits INI associated changes in cerebral blood flow, resting state activity, glutamate levels in the striatum and DLPFC, and visuospatial task performance.

### Hypotheses

We hypothesize that: 1) INI, but not INP, will change cerebral blood flow and resting state connectivity, increase glutamate levels in the striatum and DLPFC, and improve visuospatial memory performance; 2) oral (OLA), but not oral PL, will inhibit the effect of INI on these parameters. There will be no difference between INI and INP in the presence of OLA (and performance in both these arms will be worse than that in the INP-PL arm).

## Methods and materials

**Fig 1:**
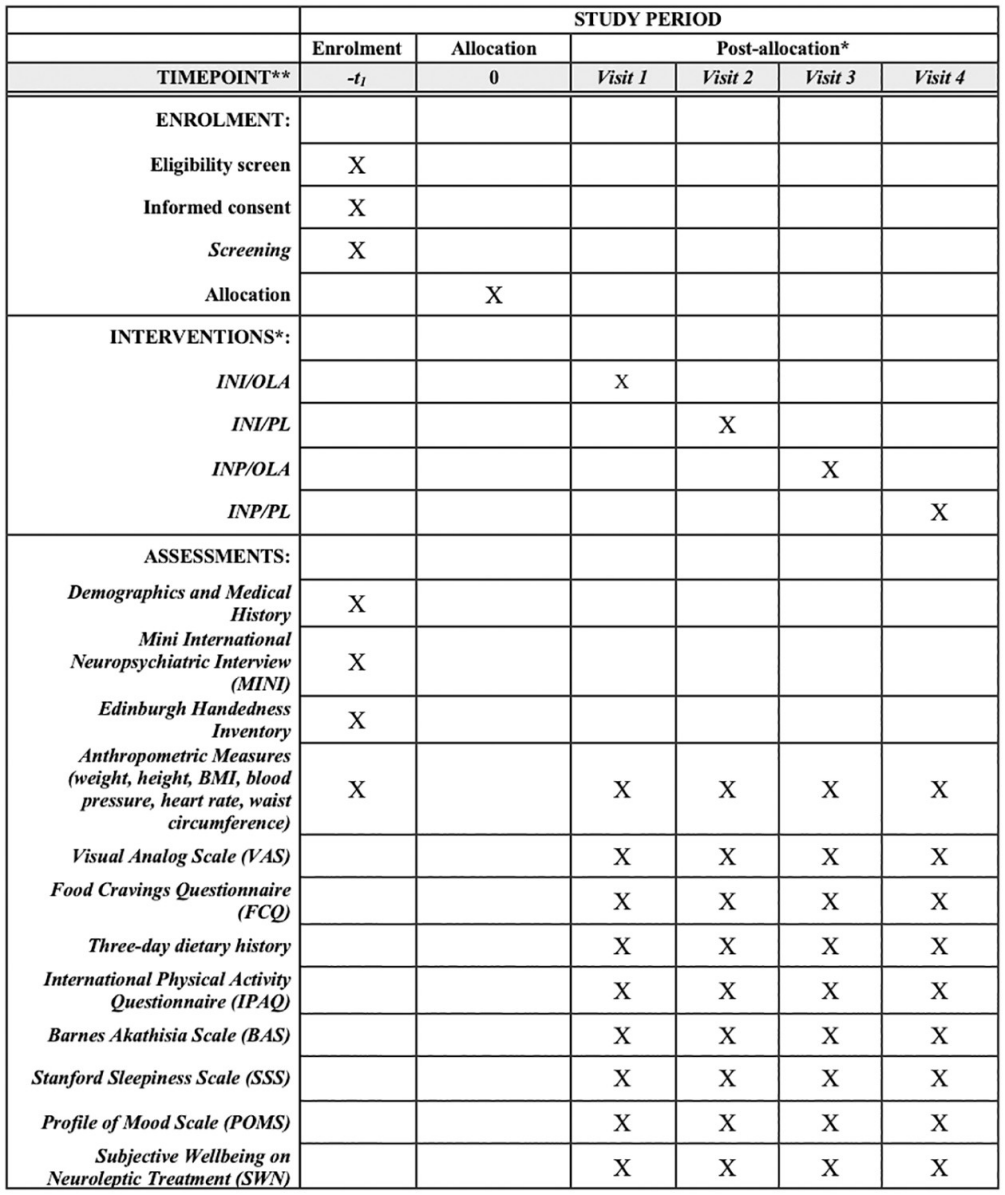
SPIRIT schedule of enrolment, interventions, and assessments. * Post-allocation sequence for order of intervention is randomized for each participant and varies across participants. Possible sequences are shown in Table 2.

**Fig 2:**
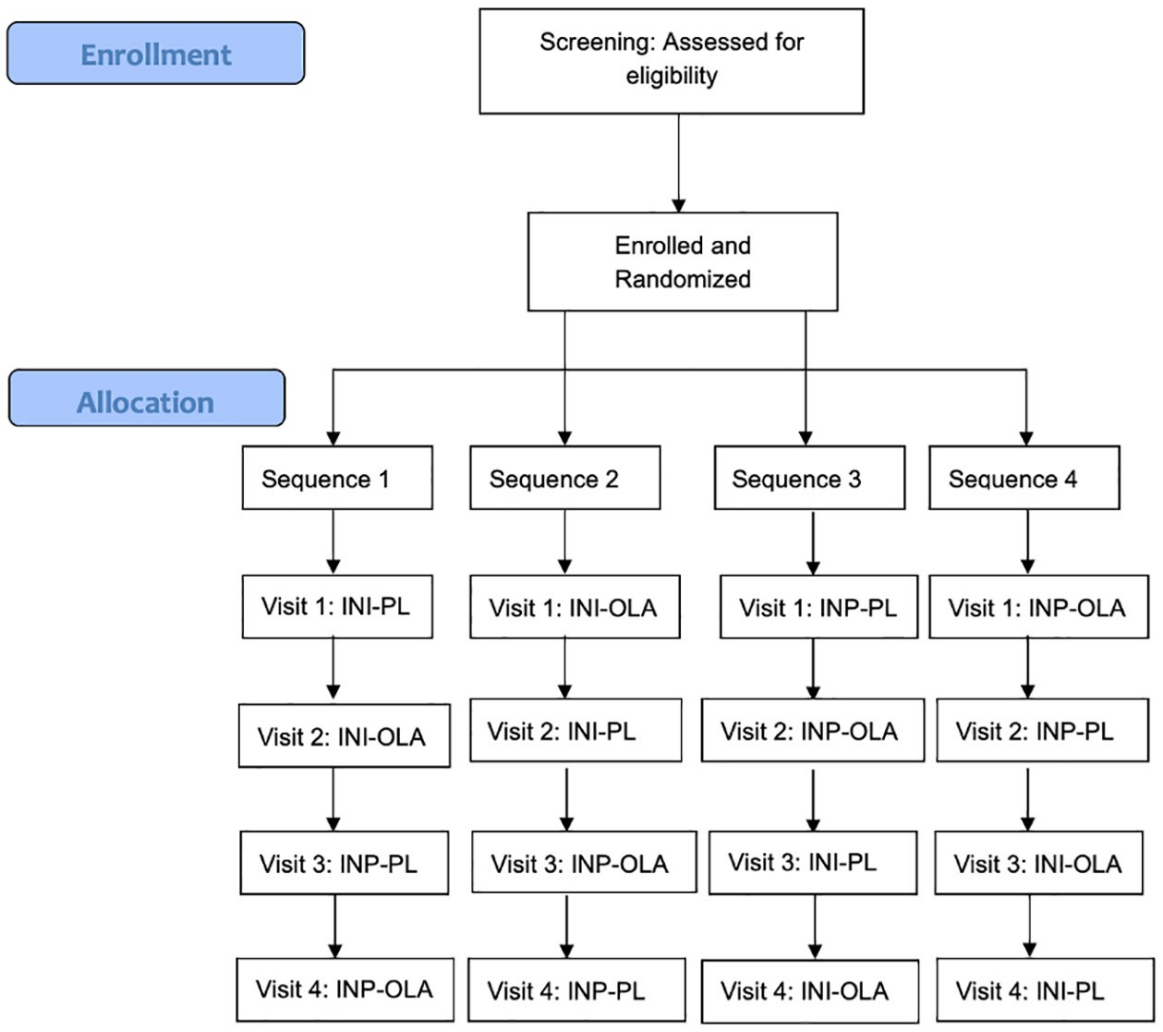
Study CONSORT flowchart.

The study is a single center randomized controlled trial (RCT) being conducted at the Centre for Addiction and Mental Health (CAMH), a psychiatric teaching hospital in Toronto, Canada. It has been granted full ethics approval by the CAMH Research Ethics Board (REB Protocol # 075/2017 Version #9, August 30, 2021; S1 File). This study is also approved under the purview of Health Canada and has been registered with ClinicalTrials.gov (NCT03741478). Funding support is being received by the Canadian Institutes of Health Research (CIHR # PJT-153262) and the Physicians’ Services Incorporated Foundation (PSI Grant #18–33). The Standard Protocol Items: Recommendations for Interventional Trials (SPIRIT) schedule of enrolment is reported in Fig 1 and the Consolidated Standards of Reporting Trials (CONSORT) flow diagram in Fig 2. The SPIRIT checklist is available in S2 File.

### Study design

**Fig 3:**
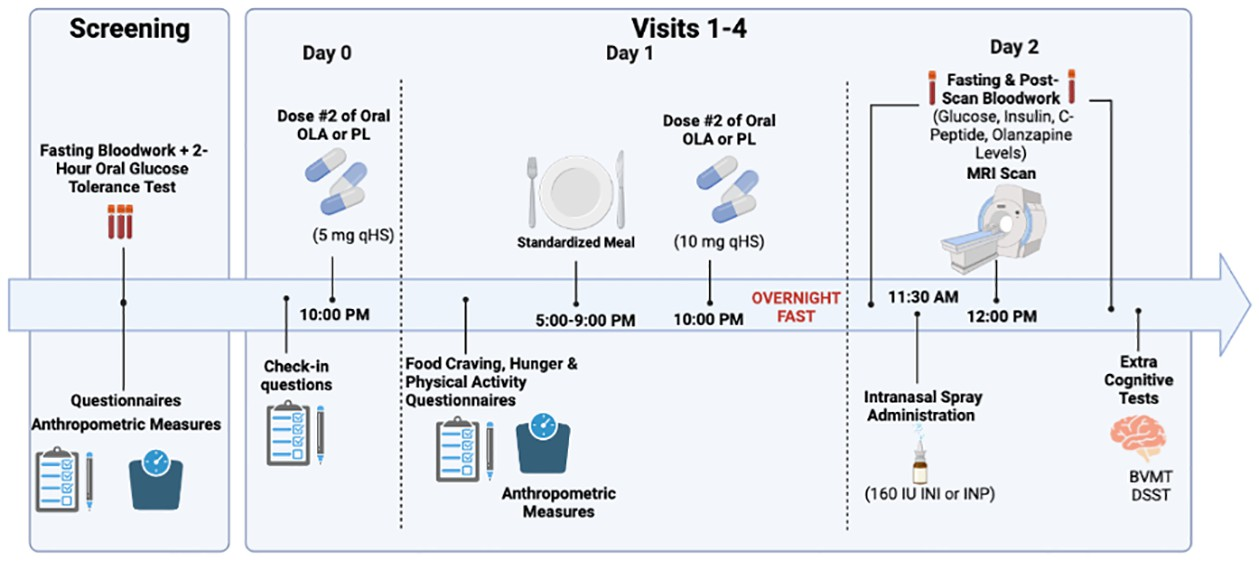
MRI and cognitive testing visit schedule.

This study will follow a single-blind, crossover design wherein each participant will receive all 4 possible treatment combinations (INP-PL, INP-OLA, INI-PL, and INI-OLA) (summarized in Table 1). These will be administered at different times in a predetermined sequence to counterbalance the effects of treatments across participants. The assignment of participants to sequences will be at random. Each participant will be investigated on 4 separate occasions (i.e., 4 study periods), 2–6 weeks apart. Each of the study periods will follow a three-day protocol (Fig 3): administration of OLA 5 mg HS (or placebo) on day 0, OLA 10 mg HS (or placebo) on day 1, and cognitive testing and MRI scanning on day 2. MRI assessments will occur 15 minutes after administering 160 International Units (IU) of INI/INP and will involve measuring cerebral blood flow, resting state activity, glutamate levels in specific regions of interest, and the spatial n-back cognitive test of visuospatial function [26]. The two acute OLA doses of 5 mg and 10 mg were chosen for reasons of practicality and safety, although this is not representative of how the drug is used clinically (i.e. daily administration, often at higher doses) [8]. Nonetheless, previous research has shown that a single 10 mg dose of OLA in healthy controls may invoke early changes in some parameters of glucose and lipid metabolism, justifying the chosen dose in this study [8]. The rationale for the chosen INI dose is based on a study finding that in nine healthy men receiving placebo, 40 IU, 80 IU or 160 IU of INI in a randomized order, there was a dose-dependent relationship on regional brain activity, with the strongest effects being observed after 160 IU [16]. It should be noted that the high dose of INI is necessary for the experimental design but likely does not reflect postprandial insulin concentration. Blood samples will also be drawn before and after the MRI to measure insulin spillover and olanzapine blood levels.

**Table 1:** Scheme of the Latin Square.

Possible Sequences	Time 1	Time 2	Time 3	Time 4
1	INI-PL	INI-OLA	INP-PL	INP-OLA
2	INI-OLA	INI-PL	INP-OLA	INP-PL
3	INP-PL	INP-OLA	INI-PL	INI-OLA
4	INP-OLA	INP-PL	INI-OLA	INI-PL

INI = intranasal insulin; INP = intranasal placebo; OLA = Olanzapine; PL = oral placebo

### Investigational products

The oral investigational agent used in this study is olanzapine (Generic brand: Apo-Olanzapine), administered at night over the course of two days (5mg and 10 mg, respectively). The intranasal investigational product will be either Insulin Lispro (Humalog) or placebo (0.9% saline solution) administered via a metered nasal dispenser (Medisca, Canada). A single spray dispenses 0.1 mL (10 IU) of insulin. Following a standardized operating protocol, participants are trained on self-administration of the intranasal spray. A single spray is administered in each nostril while inhaling. After waiting 1 minute, another spray is similarly administered in each nostril. The intranasal spray process in both nostrils is repeated for a total of 8 times (total of 160 IU will be delivered). Medication compliance will be monitored by collecting empty medication blister packs and measuring blood levels of OLA. INI or INP will be administered in the presence of study staff.

### Study participants

A total of 32 healthy non-obese volunteers with no history of psychiatric illness will be recruited to participate in this study. Participants of any race or ethnicity will be included in this study if they meet the following criteria: 1) Between the age of 17 and 45; 2) No personal or family (first degree relative) history of diabetes; 3) No history of psychiatric illness; and 4) Right-handed. Participants will be excluded for the following reasons: 1) History of psychiatric illness (assessed using the Mini International Neuropsychiatric Interview (MINI)); 2) Left-handedness; 3) Pre-diabetes or diabetes (fasting glucose ≥6.0 mmol/L or use of an anti-diabetic drug); 4) Evidence of impaired glucose tolerance on oral glucose tolerance test (OGTT); 5) Family history of diabetes (first degree relative such as a parent or sibling); 6) Use of weight reducing agents or other medications based on the discretion of the PI; 7) History of liver disease or AST > 2 times upper limit of normal; 8) History of kidney disease; 9) Major medical or surgical event within last 6 months; 10) Any condition that interferes with the safe acquisition of MRI data such as metal implants, pacemakers, cochlear implants, claustrophobia etc.; and 11) Pregnancy and/or breastfeeding. Participants can continue to receive routine medical care while enrolled.

Any participant may be discontinued from the study at the discretion of the investigators if this is deemed to be in the best interest of the participant. The decision may be made either to protect the participant’s health and safety, or because it is part of the research plan that people who develop certain conditions may not continue to participate. Reasons for withdrawing individual participants from the study may include one or more of the following: a) major protocol violation, b) participant lost to follow-up, c) withdrawal of consent or d) participant becomes pregnant or breast-feeding.

### Recruitment and trial procedures

Healthy volunteers will be recruited from REB-approved postings in the community, on social media, and through the CAMH research registry for healthy volunteers. Interested individuals will be pre-screened by study staff with the REB approved “pre-screen form” to determine preliminary eligibility. If deemed eligible based on this form, participants will be invited to complete informed consent and then begin the official screening procedures of the study. Study staff will obtain written, signed informed consent from each participant (S3 File). Only once eligibility is confirmed by the Principal Investigator (PI) will the participant be enrolled in the study. Participants will be well informed that their participation in the study is completely voluntary, and they can withdraw their consent at any time, without consequence. The study team will maintain records for the number of people approached, screened, eligible, withdrawn, and the reasons for non-enrolment. Any research information recorded for, or resulting from, participation in this research study prior to the date that the subject formally withdrew their consent will be retained and may continue to be used and disclosed by the investigators for research purposes; however, no new data will be collected.

### Randomization, blinding and allocation concealment methods

The study staff will confirm eligibility and assign a participant trial number. Eligible participants will be randomized to receiving 1 of 4 treatment sequences (outlined in Table 1). These randomized sequences are computer generated using SPSS syntax and a seed to ensure reproducibility. The assignment of treatment condition, according to the randomization table, is done by CAMH pharmacy staff who are unblinded and not involved in any other trial matters; at any time, the pharmacy staff is only unblinded to the current randomization and not the entire randomization table. Oral OLA and PL will be dispensed by the pharmacy in identical looking capsules packaged in blister packs. INI or INP will be transferred from their respective vials into an unlabeled metered spray bottle that is identical for both conditions. Study staff will not be involved in any pharmacy related processes. Treatment sequences will be unblinded on an ongoing basis, once a participant has completed all four study visits. Premature unblinding will only occur if a serious adverse event were to occur that requires knowing the allocation the participant has received.

### Study assessments

#### Screening visit

Prior to inviting participants to complete the consent form and begin the screening visits of the study, participants will be asked a series of eligibility-related questions through a telephone screen to act as an initial check of eligibility and safety. The telephone pre-screening will be reviewed by the PI and if the participant has been deemed eligible, they will proceed to the screening visits of the study for informed consent and thorough assessment of eligibility. The following demographic and clinical information will be collected to characterize the sample and confirm study eligibility: demographic variables (e.g. age, sex, race, education level/type); personal and family medical history; personal psychiatric history (assessed using the Mini-International Neuropsychiatric Interview (MINI)); anthropometric measures (e.g. blood pressure, weight, waist circumference, height, BMI); fasting bloodwork and 2-hour oral glucose tolerance test to rule out prediabetes or type 2 diabetes (using a standard glucose drink (75gm)); and the Edinburgh Handedness Inventory (EHI) to assess hand dominance or laterality. Participants will only be enrolled in the study once eligibility has been determined following the screening visit.

#### Subsequent study visits 1–4

Once eligibility is confirmed, participants will be randomized into the study to receive all 4 treatment conditions, in a crossover design. The measures acquired at each visit are summarized below.

*Day 0*: *First dose of medications*. Participants will take oral OLA 5 mg (or PL) at their home around 10–10:30pm. Check-in questions will be asked to ensure there have been no changes in health conditions/medications since the last visit.

*Day 1*: *Second dose of medications*. Participants will be provided with a standardized mixed meal around 5:00 pm that can be eaten at any time up until 9:00 pm. This meal will be followed by an overnight fast until all study procedures are completed the next day. Participants will complete questionnaires related to hunger, eating habits, and physical activity (summarized in Table 2).

**Table 2:** Description of clinical scales administered in study.

Clinical Scale	Definition	Visit
**Mini-International Neuropsychiatric Interview (MINI)**	Short structured diagnostic interview used to assess and track psychiatric illness history. The administration time of the interview is approximately 15 minutes.	Screening visit
**Edinburgh Handedness Inventory (EHI)**	Measurement scale used to establish hand dominance. Participants are asked which hand they prefer to use for a list of 15 specific activities to determine their handedness (left-handed, right-handed, no preference).	Screening visit
**Visual Analog Scale (VAS)** and **Food Cravings Questionnaire (FCQ)**	These questionnaires list a series of statements people have made about their eating habits. Participants are asked to respond how frequently those statements are true for them. Completed in fasting state and repeated in non-fasting state (standardized meal to be provided).	Visits 1–4; Day 1
**Three-day dietary history**	Complete record everything the participant has had to eat and drink for the three preceding days to our study assessments on Day 2. This is collected to compare nutrition/caloric intake across participants.	Visits 1–4; Day 1
**International Physical Activity Questionnaire (IPAQ)**	Used to assess the physical activity level of each participant. Assesses the time they have spent being physically active in the last 7 days (including walking, vigorous & moderate physical activity, and number of hours they are sedentary per day).	Visits 1–4; Day 1 or 2
**Barnes Akathisia Scale (BAS)**	Rating scale that is used to assess the severity of drug-induced akathisia (a movement disorder that makes it hard for an individual to stay still; common side effect of antipsychotic drugs).	Visits 1–4; Day 2
**Stanford Sleepiness Scale (SSS)**	Subjective rating scale used to assess sedation before cognitive assessments are completed (participants assess their sleepiness on a scale of 1–7).	Visits 1–4; Day 2
**Profile of Mood Scale (POMS)** and **Subjective Wellbeing on Neuroleptic Treatment (SWN)**	Subjective rating scales administered to evaluate changes in energy, mood, and subjective well-being induced by the study medication.	Visits 1–4; Day 2

**Fig 4:**
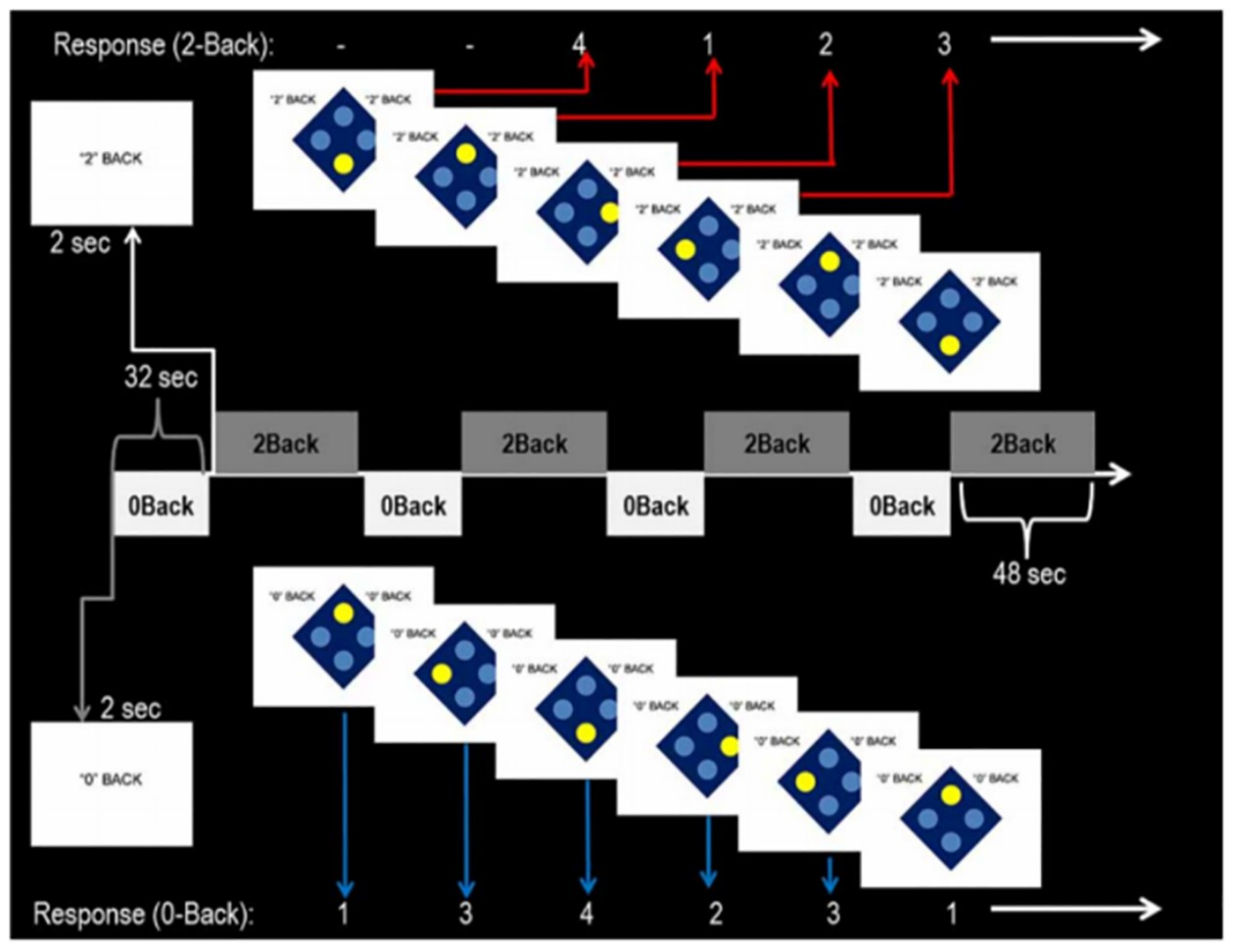
Spatial N-back fMRI paradigm.

*Day 2*: *MRI Scanning and Cognitive Tests*. Participants will be picked up by taxi and brought to CAMH for the primary study assessments to occur. Day 2 will follow the protocol outlined below:

1. Between 10:00 and 11:00 am, fasting blood samples are drawn to measure glucose, insulin, C-peptide and OLA levels.
2. Administration of 160 IU of INI (or INP) will occur ~30 minutes before the MRI scan. MRI scans will be performed using CAMH’s research dedicated 3T scanner. The MRI scanning protocol will be ~1 hour in duration and will include structural MRI, arterial spin labelling (ASL), proton magnetic resonance spectroscopy (^1^H-MRS), resting state functional MRI (fMRI), and task-based fMRI. Under resting conditions, participants will be asked to remain awake but keep their eyes closed and let their mind wander. Task fMRI data will be acquired during the performance of the spatial n-back paradigm, a visuospatial working memory task [26]. The task is a block design spatial variant of the n-back working memory task with two levels of difficulty (Fig 4). The paradigm has been designed using E-Prime (Psychology Software Tools, Pittsburgh, PA) and has been shown to activate frontal and parietal brain regions involved in spatial cognition and working memory [26]. In this continuous performance task, participants will alternate between 0 back and 2 back conditions. Participants are shown four large blue dots in a diamond-shaped diagram, indicating the four possible positions of the stimulus. The stimulus consists of one dot changing colour at a time. Participants will be instructed to respond to the stimulus by button box presses as fast and as accurately as possible. Responses are made either directly following the stimulus (0-back), or with a delay of two stimuli (2-back). Outcome measures are both accuracy and reaction time. Acquisition parameters for the MRI scan sequence are outlined in S4 File, along with additional details for the spatial n-back intrascanner cognitive task.
3. After the MRI, a second post-scan blood sample will be drawn (repeating measures of glucose, insulin, C-peptide, and OLA levels).
4. MRI assessments will also be followed by brief extra-scanner cognitive testing involving the Digit Symbol Substitution Test (DSST) and Brief Visuospatial Memory Test-Revised (BVMT) [27, 28]. Brief versions of the neuropsychological tests (i.e., which can be completed within a 2-hour period) are employed due to insulin pharmacokinetics, which appear to be sensitive relative to neuropsychological effects. In addition to advantages of brevity, the BVMT has alternate form capacity, with an associated low risk of direct practice effects during serial neuropsychological assessments. The DSST is a widely used and simple cognitive task that principally assesses visual scanning and processing speed and is sensitive to sedation [29, 30].
5. Once all study procedures are completed, participants will be compensated and provided with lunch before being discharged.

The above procedures are repeated for a total of 4 research visits. Participants will have an emergency contact card during the visits in the event of any side effects to be promptly assessed and treated, if necessary, by the study physicians.

### Description of clinical scales

A description of the clinical scales administered as part of the study, and at which visit are outlined in Table 2.

### Data analysis

#### MRI analysis

The fMRI and ASL data will be preprocessed and analyzed using appropriate tools available in SPM12 and FSL libraries. Standard preprocessing steps will be performed before analysis. Contrasts will be generated for activation differences between spatial n-back task conditions. MRS data will be analyzed using LCModel version 6.3-0E and Osprey. Spectra will be normalized to the unsuppressed water signal, permitting quantification of neurometabolic levels (i.e., glutamate) in the volume of interest (VOI).

#### Statistical analysis

The sample size of this pilot study is powered to detect moderate effect sizes. Since this is an exploratory study, data will be used to generate future sample sizes. The same set of statistical analyses will be completed for all neuroimaging parameters and behavioural data. Descriptive analysis including data visualization of individual trajectories will be conducted to inspect the data for atypical data points and to characterize the data. Mixed effect models that control for period (1, 2, 3 and 4) and sequence (1, 2, 3 and 4) and specify random intercept for subjects will be used to test all hypotheses as well as to estimate effect sizes. Hypothesis 1 will be tested by comparing INI-PL with INP-PL using linear contrasts. Hypothesis 2 will also use linear contrasts to compare the difference INI-PL—INP-PL with the difference INI-OLA—INP-OLA. Statistical significance will be declared if p-value < 0.05 and regardless of it, estimated means with their standard errors will be reported for all outcomes. Eventual dropouts will be included in the model. Since OLA is associated with sedation which may impact our cognitive domains of interest, we will quantify sedation using: a) the Stanford Sleepiness Scale (SSS) [31]; and b) a digit symbol substitution test (DSST) [29]. The SSS and DSST scores will be used as covariates to control for sedation. Scores on the POMS and SWN scales will be used as covariates to control for changes in energy, mood, and subjective well-being induced by the study medication. Age, sex, and baseline BMI will be used as additional covariates in the analysis.

### Assessment of safety

Adverse events will be recorded in the Adverse Event Log. Adverse events include any unfavorable change in a study participant’s physical or psychological wellbeing during the study period, which may or may not be the result of participation in the study. Adverse events will be reported to the CAMH REB if all of the following REB-defined criteria are met for an Unanticipated Problem: 1) The event is unexpected (relative to the product monograph, research protocol, and consent forms), 2) The event is related or possibly related to participation in the research, as judged by the PI, 3) The event suggests that the research places the participants or others at a greater risk of harm than was previously known or recognized. Required reporting will occur within 48 hours of the PI becoming aware of the event. Any Serious Unexpected Adverse Drug Reactions will be reported to Health Canada if all of the Health Canada-defined criteria are met: 1) The medical occurrence is serious (results in death, is life-threatening, requires hospitalization or prolongation of existing hospitalization, or is a congenital anomaly/birth defect), 2) The event is unexpected (nature or severity not consistent with information in the relevant source documents such as product monograph), and 3) It is judged by the reporting health care professional as a having a reasonable suspected causal relationship to the medicinal product. Where the event is neither fatal nor life threatening, required reporting will occur within 15 days of awareness of the information. If fatal or life-threatening, it will be reported within 7 days.

### Confidentiality

There is a potential risk of breach of confidentiality that is inherent in all research protocols. Breach of confidentiality will be minimized by the staff who will maintain research data (identified only by participant code number not related to name, or date of birth) in separate charts and a dedicated password protected electronic database. A list of participant names, their ID numbers, and information about how they can be reached will be kept in a separate locked cabinet with access only to study personnel authorized by the PI. Procedures have been established, and will be followed, to minimize the risk of breach of confidentiality. Procedures to maintain confidentially include: (1) formal training sessions for all research staff emphasizing the importance of confidentiality; (2) specific procedures developed to protect participants’ confidentiality, and (3) formal mechanisms limiting access to information that can link data to individual participants. All information obtained from participants will be kept as confidential as possible. Computer based files/data will be entered into password-secured databases and paper-based files will be stored in a secure location. These data will only be accessible to personnel involved in the study and they will abide by confidentiality regulations of the REB. The ethics committee will be granted direct access to the study participants’ original medical records for verification of clinical trial procedures and/or data, without violating the confidentiality of the participants, to the extent permitted by the law and regulations.

### Study management and materials

CAMH investigators will retain a participant identification code list if they need to contact participants after the study. This list will contain the complete name, identification number, address and phone number of all participants and will be held confidentially at the investigators site after completion of the study. Study data will be entered in a secure database. An eCRF/CRF will be completed for each participant enrolled in the study. A participant screening log, noting reasons for screen failure, where applicable, will be maintained for all participants. The investigator will document the obtained informed consent and record medical and psychiatric history, medications, and efficacy data in the eCRF/CRF. Clinical scales and neuropsychological assessments will be considered source documents and will be incorporated into the eCRF/CRF in a confidential manner. Study data will be entered in a secure database using Research Electronic Data Capture (REDCap) software. At point-of-entry, data values will undergo consistency edits (e.g., ID validation, range verification, duplicate detection) and personnel will be required to correct errors.

### Knowledge transmission and dissemination plans

The results from the current study will be presented at international conferences and published in open access journals to ensure widest reach to other qualified researchers and clinicians.

## Discussion

Brain insulin resistance is emerging as a shared pathological feature between cognitive and metabolic disorders. Drawing parallels between brain insulin resistance, SCZ, and its treatments, the illness is associated with high rates of type 2 diabetes, obesity and impairments in cognitive function which overlap with those linked to metabolic disease and insulin resistance. Of interest, cognitive deficits are understood to be a key illness domain of SCZ for which no effective pharmacological interventions have been established. Thus, the possibility exists that AP efficacy to target cognitive deficits may be diminished secondarily via AP-induced metabolic dysregulation and insulin resistance. Examination of the translational value of our earlier findings suggesting OLA can induce central resistance to insulin is both intriguing and very clinically relevant to domains of physical health and, possibly, psychopathology.

This study aims to enhance our understanding of the mechanisms behind metabolic adverse effects of AP drugs and their cognitive effects. In an exploratory fashion, this study will employ an MRI-based assay of brain insulin resistance to investigate the potential cognitive impact of APs in the context of central insulin stimulation. Hence, this study can help shed light on how insulin resistance in the brain can affect neuroimaging, cognitive, and metabolic outcomes. Additionally, this work is of relevance to all diseases positioned at the crossroads of metabolic and cognitive health. It has the potential to initiate new streams of work in an area of significant unmet need that intercepts disciplines of physiology, endocrinology, and psychiatry, and may lead to new therapeutic strategies. Specifically, evidence of AP-induced brain insulin resistance may advise use of adjunctive treatment with targeted central insulin sensitizers to address metabolic and possibly cognitive dysfunction at the earliest stages of the illness.

### Limitations and measures to minimize bias

It is recognized that the single-blind nature of this study is a limitation. However, it should be noted that study staff will operate in a double-blind fashion while participants are actively enrolled. In this regard, blind assignments will be kept by the CAMH pharmacy and only released once a participant completes all four visits of the study to be included in the ongoing analysis. A pharmacy member external to the study team will have access to unblinding codes in case of a serious adverse event. The trial will be registered with a publicly available, free-to-access, searchable clinical trial registry before approaching the first participant to avoid bias selection of the reported result(s). Furthermore, study staff administering and scoring the extra-scanner cognitive assessments will be blinded to the INI/INP allocation to minimize any bias (they will not be involved in transferring the product into the nasal spray bottle).

## Supporting information

S1 File Clinical study protocol. (DOCX)

S2 File SPIRIT checklist. (DOC)

S3 File Informed consent form. (DOCX)

S4 File MRI acquisition parameters. (DOCX)

S5 File (PDF)
