# Supplementary material for: Investigating the effects of antipsychotics on brain insulin action: Study protocol for a multi-modality magnetic resonance imaging (MRI) study in healthy controls
Source: PLoS One. 2022 Nov 28;17(11):e0277211. doi: 10.1371/journal.pone.0277211 (PMC9704670; doi:10.1371/journal.pone.0277211)
Supplement: S1 File — (DOCX) [file pone.0277211.s001.docx]

##### STUDY PROTOCOL

**Study Title:** Effect of antipsychotics on central insulin action in relation to glucose metabolism and cognition in healthy volunteers

**REB#:** 075/2017

**Principal Investigators:** Dr. Margaret Hahn MD, PhD, FRCPC

Institution: Centre for Addiction and Mental Health

**Co-Principal Investigator:** Dr. Satya Dash MD, PhD, FRCPC, MRCP (UK)

**Co-Investigators:** Dr. Sri Mahavir Agarwal MBBS, MD

Dr. Adria Giacca MD

Dr. Ariel Graff-Guerrero MD, PhD, FRCPC

Dr. Daniel Mueller MD, PhD, FRCPC

Dr. Gary Remington MD, PhD, FRCPC

Dr. Aristotle Voineskos MD, PhD, FRCPC

Dr. Valerie Taylor MD,PhD, FRCPC

**Source of funding:** Canadian Institute of Health Research (CIHR)

#### Statement of Objectives and Hypotheses

- 1. **Objectives**

1. To examine whether a single dose of olanzapine (OLA) given to young healthy volunteers during pancreatic euglycemic clamps can inhibit the ability of a central insulin stimulus (i.e. intranasal insulin administration) to reduce endogenous glucose production (EGP).
2. In an exploratory aim, we will examine whether a single dose of OLA can inhibit improvements associated with intranasal insulin administration on specific cognitive domains (visuospatial, and verbal memory). We will also explore the effects of insulin and OLA on neurochemical and neurohemodynamic measures.

The study will have two separate and parallel arms. The first arm will test the first objective while the second arm will examine the second objective. Participation in one arm does not exclude participation from the other, provided sufficient wash out period between the 2 arms is maintained (>1 week).

**1.2 Hypotheses**

1. Primary hypothesis (Glucose metabolism: Pancreatic euglycemic clamp experiments):

Intranasal insulin (INI) will be associated with a decrease in **endogenous** **glucose production** (EGP) (our primary outcome measure) relative to intranasal placebo (INP). This effect of glucose production lowering by INI will be lost if OLA is co-administered. In the absence of INI administration, there will be no difference between OLA and oral placebo (PL). We do not expect any of the treatments to differ with respect to glucose disposal (Gd). Predicted differences between treatments in Dextrose Infusion Rate (InfR) reflect differences in EGP. In summary, we predict the following treatment effects **relative to the INP-PL group** (i.e. no active treatment) (Table 1):

| Treatments | | Measures derived from Pancreatic clamp procedure | | |
| --- | --- | --- | --- | --- |
| Intranasal | Oral | Dextrose InfR | EGP | Gd |
| INI | PL | 🡹 | 🡻 | 🡺🡸 |
| INI | OLA | 🡺🡸 | 🡺🡸 | 🡺🡸 |

1. Secondary hypothesis (Cognition):

OLA will be associated with decrements in cognitive measures as compared to PL. Further, OLA will block the beneficial effect of INI on cognition. Hence, there will be no difference between the INI and INP in the presence of OLA (and performance in both these arms will be worse than that in the INP-PL arm). In summary, we predict the following treatment effects **relative to the INP-PL group** (Table 2):

| Treatments | | Cognition:  (visuospatial memory and verbal memory) |
| --- | --- | --- |
| Intranasal | Oral |  |
| INI | PL | 🡹 |
| INP | OLA | 🡻 |
| INI | OLA | 🡻 |

- 1. We also hypothesize that INI will result in adaptive changes in neurochemical and neurohemodynamic measures as studied using MRI techniques. Furthermore, OLA will block these effects of INI.

#### Background/Rationale

***i) Antipsychotic (AP) use and metabolic adverse effects in youth***

Antipsychotic (AP) medications are currently the cornerstone of treatment for schizophrenia (SCZ), with off-label prescription rapidly increasing in youth and adolescent populations (1, 2) (**Figure 1**). However, APs, particularly so-called “second-generation agents”, have been associated with serious metabolic adverse effects including weight gain and type 2 diabetes (T2D), in a population with an established 2-fold increase in standardized mortality ratio attributable to cardiovascular disease (3). It is increasingly becoming clear that children and adolescents are especially vulnerable to these metabolic adverse effects, and that one of the best established risk factors for AP-induced weight gain is lack of previous exposure to these medications (4-6). Recent data also suggests that young individuals treated with these agents are at high risk of glucose dysregulation; studies involving patients under 30 who switched to or started treatment with higher metabolic risk APs have shown concerning rates of glucose intolerance (55%) or impaired fasting glucose levels (7, 8). Cardiometabolic data in first episode patients (ages 14-40) with less than 6 months of exposure to APs (recently published in JAMA Psychiatry) found rates of prediabetes exceeding 15%, and significant associations between AP treatment and insulin resistance (9). Similarly, a recent meta-analysis, also published in JAMA, confirmed that cumulative risk and exposure adjusted incidences of T2D were significantly higher in AP-exposed youth as compared to healthy and psychiatric controls (10). Beyond the devastating medical sequelae of obesity and diabetes, AP-associated metabolic adverse effects have also been reported to cause medication non-compliance, low self-esteem, and decreased quality of life (11, 12).


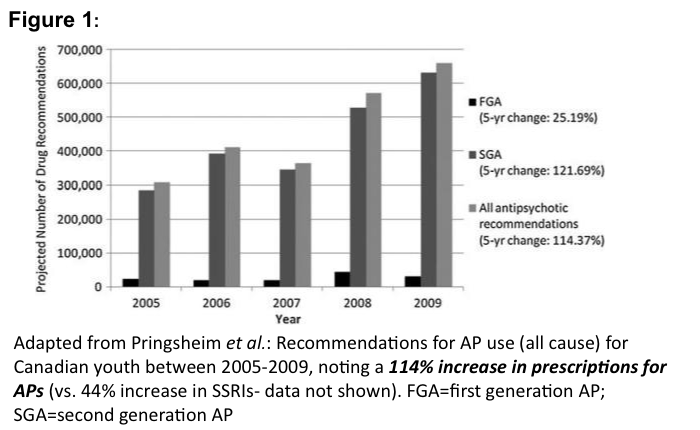


***ii) Mechanisms underlying glucose dysregulation in psychosis*** *(*see **Figure 2***)*

Though these metabolic effects are concerning, mechanisms underlying AP effects on glucose metabolism are largely undetermined. AP-induced weight gain is a well-established risk factor for T2D, and accounts in part for the high prevalence of diabetes in SCZ. The picture is also complicated by illness-related factors, including lifestyle (i.e. high smoking rates, poor dietary habits), and inherent links between illness biology and metabolic dysfunction(13, 14). Moreover, an examination of the relationship between APs and T2D has indicated that factors independent of AP-induced weight gain contribute to this risk. Data from animal models, including our work, has shown that a single dose of APs including risperidone, quetiapine, clozapine and olanzapine (OLA) reliably cause acute, profound perturbations in glucose metabolism, with evidence pointing to the liver as the target organ for the drug-induced perturbations (15-17). More recent work by our group and others also points towards the brain as a potential modulator of these AP-induced side effects(18-21). Importantly, weight-gain independent effects on glucose metabolism observed in rodents have been replicated in healthy humans (including by our group), where anywhere from 1-9 days of dosing with OLA (and interestingly also aripiprazole - an AP agent considered to be metabolically neutral) demonstrate perturbations in glucose metabolism(22-26). This supports that: **1**) APs induce glucose dysregulation through “direct” molecular mechanisms even before weight gain occurs; **2**) these effects can occur in the absence of additional risk added by the illness (state and trait); and **3)** moving from animal models to humans is a viable approach.


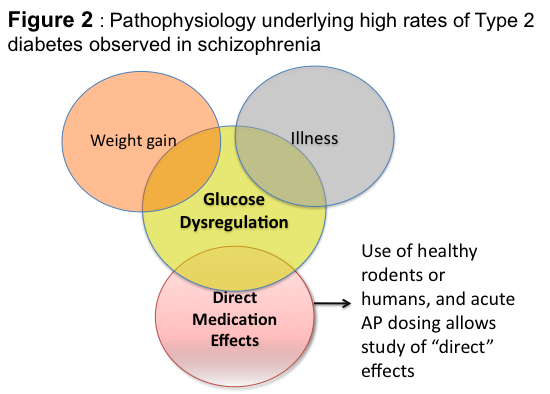


***iii) Schizophrenia (SCZ), insulin and APs: implications for metabolism and psychopathology***

Given the complex relationship between the illness of SCZ, treatments with APs, and underlying metabolic vulnerability, the question of whether brain insulin signaling perturbations could represent a link between psychopathology and metabolic dysfunction has emerged (27). Insulin receptors are expressed at high levels in many brain areas and cell types, and are now understood to play a significant role in neuronal growth, and memory; abnormalities in these pathways have been found in patients with SCZ (27). At the same time, there is growing evidence of AP effects on brain insulin pathways (27). Linking disruptions of insulin signaling in SCZ to metabolic dysregulation, the brain is an insulin-sensitive organ that regulates food intake, lipid, and glucose homeostasis. Insulin, separate from its effects on liver receptors, regulates hepatic glucose production through insulin receptors in the brain; it is well established that an intracerebroventricular (ICV) infusion of insulin in rodents results in a decrease in endogenous glucose production by the liver (with no effect on glucose disposal)(28, 29). Increased endogenous glucose production is the major source of fasting, and post-absorptive hyperglycemia in T2D(30).

Translating these findings to humans, a recent study published by our co-principal applicant (S.Dash) examined acute administration of intranasal insulin (analogous to ICV infusions in rodents) in healthy male volunteers, and noted a significant decrease in endogenous glucose production (with no effect on glucose disposal) during a pancreatic euglycemic clamp(31). Central insulin also impacts processes other than regulation of hepatic glucose production: 1) insulin possesses anorexigenic effects in males; 2) reduces intake of palatable foods in females; 3) and can modulate food-related activity in the CNS(32). Thus AP-induced central insulin resistance can have other metabolic implications beyond dysregulation of hepatic glucose production, a hallmark of T2D. In addition, intranasal insulin has repeatedly been shown to improve varying aspects of cognition, most consistently verbal memory and visuospatial function (in males and females)(32, 33), also impaired in SCZ(34). However, three available studies in SCZ patients have examined intranasal insulin to target AP-induced weight gain(35) or core cognitive illness deficits(36, 37), and failed to note benefits. This brings up the possibility that factors related to the illness itself (obesity, or illness biology linked with defective insulin signaling in the brain), and/or direct AP-induced disruptions in insulin signaling, could explain lack of effects of intranasal insulin, which are otherwise observed in healthy controls(32). The use of healthy rats or AP-naive, healthy volunteers (avoiding the effects of illness), and acute dosing, i.e., a single dose (to avoid weight gain), allows us to dissect whether APs can directly impede glucoregulatory and/or cognitive effects of central insulin (**Figure 2**).

***iv) Preliminary evidence of AP-induced disruptions in central insulin sensing in rodents***

Based on our well established rodent model demonstrating that OLA can acutely induce hepatic insulin resistance, we set out to explore whether this could be occurring via defective insulin sensing at the level of the brain. We used healthy rats (avoiding confounding effects of illness) which were given an acute injection of OLA (to avoid adiposity changes). In our previous work, we had employed hyperinsulinemic euglycemic clamps (HIEC) (15, 38), which we elected to avoid in the present paradigm. The HIEC involves a high dose of intravenously infused insulin which continuously reaches the brain (while glucose levels are kept constant at fasting levels). Thus insulin effects are *not* limited to the brain (and stimulate most tissues in the body), making dissection of peripheral and central effects difficult. Here we used the so called “pancreatic euglycemic clamp procedure” to assess glucose metabolism. During a pancreatic clamp, a peripheral infusion of somatostatin blocks endogenous production of insulin, which is then replaced at “basal” levels *via* a peripheral insulin infusion. Replacement of insulin within physiological “basal” levels allows separate manipulation of central insulin concentrations via an ICV infusion. A central insulin infusion, provided there is no leakage into the circulation, isolates central from peripheral insulin effects. During the pancreatic clamp an exogenous infusion of glucose is adjusted to maintain euglycemia in the animal. The amount of glucose an animal requires to maintain euglycemia (glucose infusion rate), is a measure of whole body insulin sensitivity. A tracer infusion allows for separate determination of glucose production and glucose utilization. Under these conditions, with no evidence of ICV insulin leakage into the periphery, we found that OLA acutely blocked the ability of central insulin to suppress endogenous glucose production by the liver (with *no effect* on glucose disposal), providing, for the first time, evidence that OLA impairs central insulin action, independently of changes in adiposity. Another interesting observation was that there was no effect of OLA on glucose production in the absence of a central insulin stimulus during clamp conditions. (See **Figure 3**, *manuscript in preparation*).


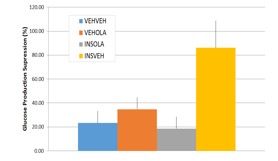


**Figure 3: Preliminary data** Olanzapine (OLA) abolishes suppression of hepatic glucose production by central insulin (INS), measured using pancreatic euglycemic clamps in Sprague-Dawley rats. Graph shows suppression hepatic glucose production (%) during steady state clamp relative to basal. p<0.05 all groups vs. INS-Vehicle (VEH). There was no effect of OLA on the rate of glucose disposal (data not shown).

***vi) Translation of findings into humans***

Brain insulin resistance is emerging as a shared pathological feature of metabolic and cognitive disturbances in obesity, T2D, and dementia. Drawing parallels between brain insulin resistance, SCZ and its treatments, the illness is associated with high rates of T2D, obesity, and impairments in cognitive function which overlap with those linked to metabolic disease and insulin resistance (39, 40). Of interest, cognitive deficits are understood to be a key illness domain of SCZ, for which no effective pharmacological interventions have been established(41). Thus, the possibility exists that AP efficacy to target cognitive deficits may be diminished secondarily via AP-induced weight gain and insulin resistance. In addition, given the role of central insulin in cognition, it is tempting to speculate that if indeed APs induce central insulin resistance, this may be a “direct” mechanism which limits improvements in cognitive functioning in patients. Thus, an examination of the translational value of our findings suggesting OLA-can induce central resistance to insulin is both intriguing and very clinically relevant to domains of physical health, and possibly psychopathology.

We now have at our disposal a paradigm, employing pancreatic euglycemic clamps in healthy humans demonstrating substantial reductions in endogenous glucose production (mostly a measure of hepatic glucose production), with no changes in glucose disposal, following intranasal insulin administration in healthy males.(31) Intranasal insulin enters the nasal cavity, bypassing the blood-brain barrier, and directly enters the CNS (42), which mimics ICV insulin administration in rodents. Thus, we can propose work, analogous to our rodent model, to examine if OLA can inhibit central (i.e. intranasal) effects of insulin in humans. This also builds on our work establishing that a single dose of OLA in healthy volunteers causes acute perturbations in glucose homeostasis (including elevated fasting glucose levels, likely indicative of OLA-induced increases in endogenous glucose production)(25). Use of young healthy volunteers holds the advantage of: **1)** avoiding confounding illness effects on glucose metabolism; and **2)** represents a population representative of AP-naïve patients who are understood to be most vulnerable to AP-induced metabolic side-effects. Acute dosing of OLA avoiding adiposity change allows us to dissect whether APs can directly inhibit central insulin action in humans.

1. **Research Study Design/Methods**
   1. **Study Duration**

The estimated length of time needed to complete the entire study (from enrolment of the first participant to completion of the last participant) is two years.

- 1. **Study Design and Assessments**

The study will have two separate and parallel arms, conducted in healthy volunteers The first arm will test the metabolic effects of INI and OLA while the second arm will examine the cognitive and neurohemodynamic and neurochemical effects. The metabolic arm will follow a single blind, crossover design wherein all participants will receive each of 3 possible treatments (INP-PL, INI-PL and INI-OLA). The cognitive arm will follow a single-blind, crossover design wherein all participants will receive each of 4 possible treatments (INP-PL, INP-OLA, INI-PL, and INI-OLA). These will be administered at different times in a predetermined sequence to counterbalance the effects of treatments across participants. The assignment of participants to sequences will be at random.

**Metabolic arm:** Each participant will be investigated on 3 separate occasions (i.e. 3 study time periods), 4-6 weeks apart (with leeway of 3 days). Each of the study periods will involve administration of OLA 5 mg HS (or placebo) on day 0, OLA 7.5 mg HS (or placebo) on day 1, and OLA 10 mg (or placebo) and the pancreatic clamp on day 2.

**Cognitive and neuroimaging arm**: Each participant will be investigated on 4 separate occasions (i.e. 4 study time periods), 2-6 weeks apart (with leeway of 3 days). Participation in one arm does not exclude participation from the other, provided sufficient wash out period between the 2 arms is maintained (>1 week). Each of the study periods will involve administration of OLA 5 mg HS (or placebo) on day 0, OLA 10 mg HS (or placebo) on day 1, and cognitive testing and MRI scanning on day 2.

**Prescreening and Screening Measures:** Prior to inviting participants to complete the consent form and begin the screening and post-screening visits of the study, participants will be asked a series of eligibility-related questions through a “telephone screen” to act as an initial check of eligibility and safety. Upon completion of the telephone screening, the screening will be reviewed by the PI and if the participant has been deemed eligible, they will proceed to the screening visits 1 and 2 of the study. To reduce burden, participants will be given the option to combine their screening and post-screening visits into one appointment, being informed that they will need to remain fasting for approximately 10 hours prior to the bloodwork start, which is in accordance with traditional fasting requirements. Participants will only be enrolled upon completion of the “Screening Visit 2”. Activities and assessments completed at Screening Visit 1 are: Informed Consent/Assent, Demographics form, medical history, MINI, Edinburgh Handedness Questionnaire (cognitive arm only), anthropometric measures (weight, height, waist circumference, blood pressure and heart rate). The following assessments may occur at screening visit 1 or 2: Oral glucose tolerance test (OGTT), fasting bloodwork, urine sample (drug screen + HCG). If the participant qualifies for the study, they will pick up their medication prior to or on Day 0 of the study procedure.

1. ***Pancreatic euglycemic clamp*** (**Days 0-2**) (**Figure 4**):

On **Day 0**, participants will take OLA 5 mg (or placebo) at their home at bedtime.

On **Day 1**, participants will be admitted to the Centre for Addiction and Mental Health (CAMH) (to our reserved research bed) or at a nearby hotel if beds are not available, provided a standardized mixed meal at around 5 P.M, (The meal can be eaten by the participant at any time up until 7:00 pm, to allow a period of at least 12 hours of fasting prior to clamp initiation the following morning). This meal will be followed by a fast and overnight stay. OLA 7.5 mg (or placebo) will be taken by the participant at bedtime. The participants will be admitted to ensure participant availability early in the morning for the clamp and tests, and to provide a standardized milieu before the test. Per PI discretion, the participant may have the option of staying at home on this night (for example, if the participant is not comfortable staying at a hotel such as due to due covid-related concerns, and they live within a reasonable distance where they can be picked up by a taxi in the morning).

On ***Day 2***, at 6AM, they will be transported to the Toronto General Hospital (TGH):

- At approximately 7AM (7-9AM) (t=-120 minutes), the pancreatic euglycemic clamp (total duration 480 minutes) will be initiated. Glucose production is quantified via a primed constant infusion of D2-glucose (dextrose infusion) (22.5 mmol/kg bolus followed by 0.25 mmol/kg/min) administered for the duration of the clamps.
- To maintain “pancreatic clamp” conditions, an IV infusion of somatostatin (30µg/hour) is initiated at t= -120 min (i.e. 7AM) to inhibit insulin, glucagon and growth hormone secretion, and concurrently replaced by a basal rate infusion of these hormones: insulin (Humulin R, Eli Lilly Canada) at 0.05units/kg/min; human recombinant growth hormone (Humatrope, Eli Lilly Canada) at 3ng/kg/min; and glucagon (Eli Lilly Canada) at 0.325ng/kg/min.
- A 20% dextrose solution, is administered as necessary to maintain euglycemia (where infusion rate represents a measure of whole body insulin sensitivity).
- At t=0 (i.e. 9AM), the participants receive 40 IU of intranasal insulin (INI) lispro, a dose which has previously been shown to suppress endogenous glucose production (by 35% relative to placebo (PL)) ^23^, or intranasal placebo (INP) via a metered nasal dispenser.
- As per established protocol, because of some spillover of 40 IU of INI lispro, volunteers during the INP visit will receive an IV infusion of insulin lispro 0.005IU/kg (or the diluent equivalent during an INI visit) over 30 minutes starting at the same time as intranasal administration.
- OLA (10mg) or PL is also administered at t=0, with timing of OLA based on pharmacokinetics, and peak plasma levels occurring within 4-6 hours, and on our single dose protocol in healthy volunteers demonstrating acute changes in glucose metabolism^18^.
- Blood samples are drawn every 30 min after the start of the clamp (t=-120 min to t=0 min), and every 10 minutes after intranasal insulin administration (t=0 to t= 360 min). The total amount of blood taken will be approximately 250 ml over the course of 6 hours.
- Blood is centrifuged immediately, and plasma stored at -20 C for further analysis. For long-term storage (later analysis), plasma will be stored at -80 C.
- OLA levels, free fatty acids, triglycerides, insulin, c-peptide, glucagon, GLP-1 and catecholamines will be measured via the central laboratory of TGH.
- The clamp procedure ends at 3pm (t=360 min), and participants are provided food, monitored for the remainder of the day (i.e. monitoring for blood pressure and sleepiness for a minimum of 30 min post-clamp) and sent home on the evening of ***Day 2***.

***
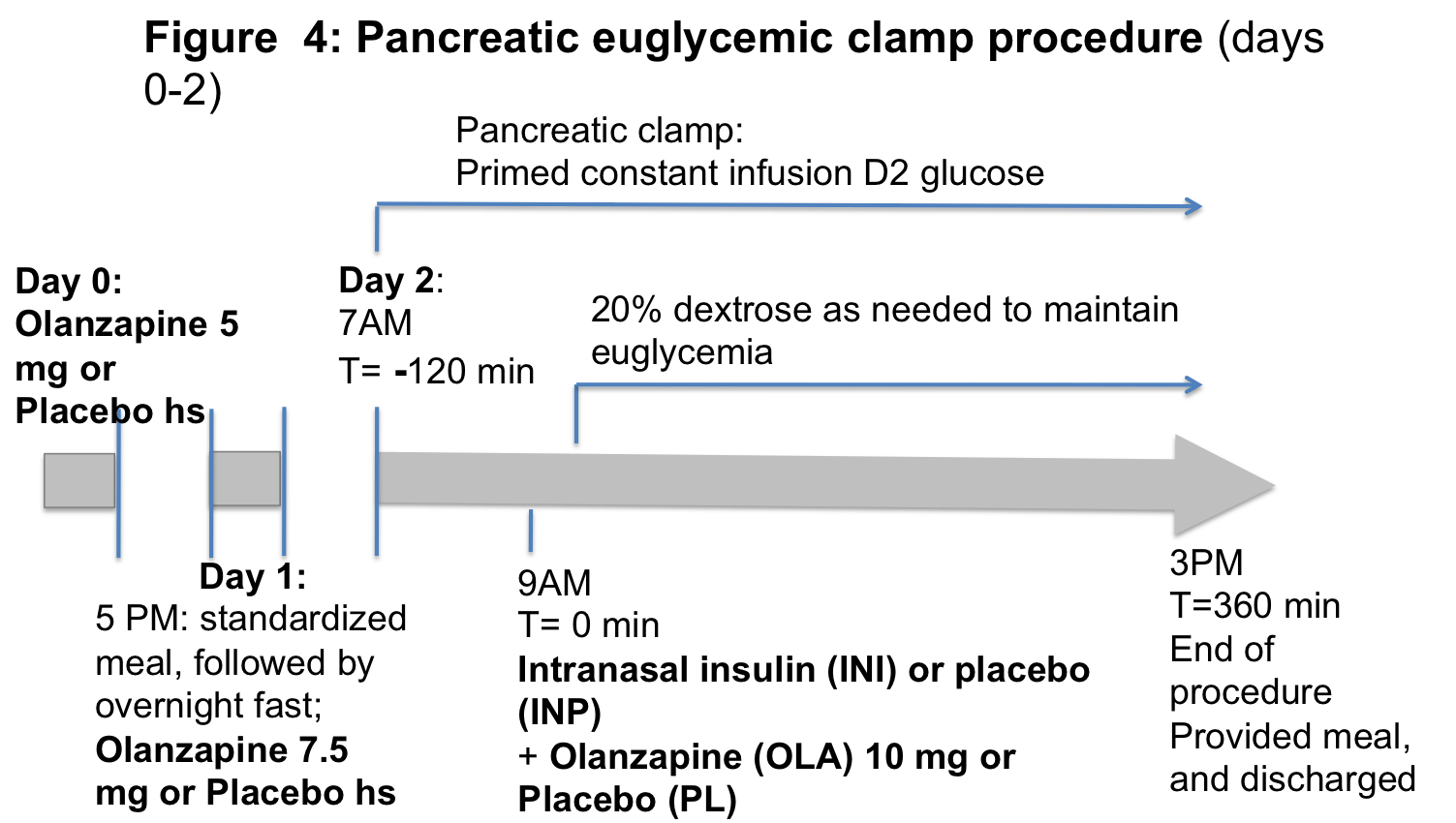

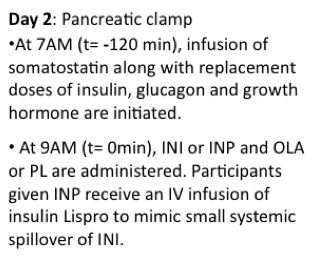
***

1. ***Cognitive Testing and MRI*** (**Days 0-2*) (*Figure 5*)*:**

On **Day 0**, participants will take OLA 5 mg (or placebo) at their home.

On **Day 1**, participants will be admitted to the Centre for Addiction and Mental Health (CAMH) (to our reserved research bed) or at a nearby hotel if beds are not available, provided a standardized mixed meal at around 5 P.M followed by OLA 10 mg (or placebo). The meal can be eaten by the participant at any time up until 9:00 pm. This meal will be followed by a fast and overnight stay. The participants will be admitted to ensure participant availability in the morning for the blood and cognitive tests, and to provide a standardized milieu before the tests. Per PI discretion, the participant may have the option of staying at home on this night (for example, if the participant is not comfortable staying at a hotel such as due to due covid-related concerns, and they live within a reasonable distance where they can be picked up by a taxi in the morning).

On ***Day 2:***

- At (10AM- 11AM), fasting samples are drawn (for glucose, insulin, c-peptide, lipids, free fatty acids, glucagon, GLP-1 and OLA levels) at CAMH.
- 160 IU of INI (or placebo) is administered at about 11 AM (11:00am to 12:00pm)
- Participants will then participate in an MRI scanning protocol at CAMH (~1 hr long) which will involve the following:
- Structural MRI (~5 min): T1-weighted, 3D magnetization prepared rapid gradient echo imaging sequence that optimizes gray white matter contrast will be acquired.
- Resting state fMRI (~ 6 min): BOLD-[Blood Oxygen Level Dependent] sensitive echo-planar imaging will be obtained.
- Test for visuospatial cognitive skills (~9 min): A functional MRI sequence will be obtained l while participants perform a cognitive task testing visuospatial skills within the scanner.
- Test for verbal memory skills (~8 min): A functional MRI sequence will be obtained while participants perform a cognitive task testing verbal memory skills within the scanner.
- Arterial spin labeling (~4 min): ASL images will be acquired to measure blood perfusion.
- Magnetic Resonance Spectroscopy (~8 min): Single voxel spectra were acquired a volume of interest (VOI) placed over frontal regions of interest.

All sequences will be optimized further in consultation with physicists after obtaining pilot data.

- MRI will be followed by brief extra-scanner cognitive testing involving the following
  - Digit symbol substitution test (DSST)
  - Profile of Mood Scale
  - Subjective Wellbeing on Neuroleptic Treatment scale

Optional cognitive tests to be completed if time permits include:

- - Brief Visuospatial Memory Test Revised
  - Verbal fluency measures (FAS, Category and Switching conditions) of the Kaplan Function System (if time permits)
- Blood work is repeated at time of completion of cognitive tasks (for glucose, insulin, c-peptide, and OLA levels).
- Brief versions of the neuropsychological tests (i.e. which can be completed within a 2-hour period) are employed due to insulin pharmacokinetics, which appear to be sensitive relative to neuropsychological effects. In addition to advantages of brevity, these test versions have alternate form capacity, with an associated low risk of direct practice effects during serial neuropsychological assessments(43, 44).
- The participants may undergo only the MRI session or the extra-scanner tests or both depending on feasibility and participant and MRI slot availability.
- Because OLA is associated with sedation which may impact our cognitive domains of interest, we will quantify sedation using: a) the Stanford sleepiness scale (SSS) (45); and, b) a digit symbol substitution test (DSST) (46). The DSST is a widely used and simple cognitive task that principally assesses visual scanning and processing speed, and is sensitive to sedation (46, 47). The SSS and DSST scores will be used as covariates to control for sedation.


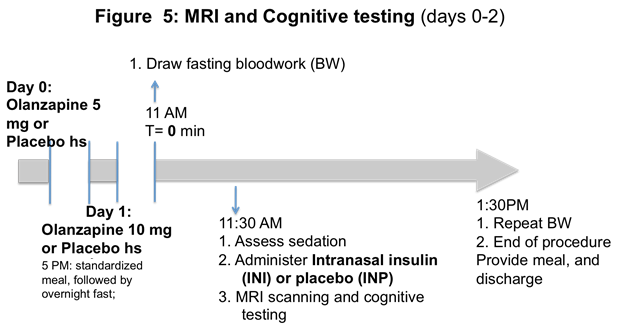


Clinical Scales:

The following scales will be used to assess participants at various time points:

- **Mini-International Neuropsychiatric Interview (MINI)**: Short structured diagnostic interview used to assess and track psychiatric illness history. The administration time of the interview is approximately 15 minutes. (Screening visit).
- **Edinburgh Handedness Inventory (EHI):** Measurement scale used to establish hand dominance. Participants are asked which hand they prefer to use for a list of 15 specific activities to determine their handedness (left-handed, right-handed, no preference. (Screening visit cognitive arm).
- **Visual Analog Scale (VAS)** and **Food Cravings Questionnaire (FCQ):** These questionnaires list a series of statements people have made about their eating habits. Participants are asked to respond how frequently those statements are true for them. Completed in fasting state and repeated in non-fasting state (standardized meal to be provided). (Completed on Day 1 (overnight stay) of each Visit in cognitive and metabolic arms).
- **3-day dietary history:** Complete record everything the participant has had to eat and drink for the three preceding days to our study assessments on Day 2. This is collected to compare nutrition/caloric intake across participants. (Completed on Day 1 of each Visit in both study arms).
- **International Physical Activity Questionnaire (IPAQ) or Simple Activity Questionnaire (SIMPAQ):** Used to assess the physical activity level of each participant. Assesses the time they have spent being physically active in the last 7 days (including walking, vigorous & moderate physical activity, and number of hours they are sedentary per day). (Completed on Day 1 or Day 2 of each Visit in both study arms).
- **Barnes Akathisia Scale (BAS):** Rating scale that is used to assess the severity of drug-induced akathisia (a movement disorder that makes it hard for an individual to stay still; common side effect of antipsychotic drugs). (Day 2 of each Visit in both study arms).
- **Stanford Sleepiness Scale (SSS):** Subjective rating scale used to assess sedation before cognitive assessments are completed (participants assess their sleepiness on a scale of 1-7). (Day 2 of each Visit in cognitive arm).
- **Profile of Mood Scale (POMS)** and **Subjective Wellbeing on Neuroleptic Treatment (SWN)** are subjective rating scales administered to evaluate changes in energy, mood, and subjective well-being induced by the study medication. (Day 2 of each Visit in cognitive arm).

Other:

Demographic information, medical history (personal and family), anthropometric measures (blood pressure, weight, waist circumference, height, BMI)

- 1. **Schedule of Procedures**
     1. **Metabolic arm**

| **Assessments** | **Week** | **0*** | **1*** | **2**** | **6**** | **10** |
| --- | --- | --- | --- | --- | --- | --- |
|  | **Visit** | **Screening Visit 1** | **Screening Visit 2** | **1** | **2** | **3** |
| Informed Consent/Assent | |  |  |  |  |  |
| Demographics form | |  |  |  |  |  |
| Eligibility/Termination Checklist | |  |  |  |  |  |
| MINI | |  |  |  |  |  |
| Anthropometric Measures (weight, height, waist circumference) | |  |  |  |  |  |
| Oral glucose tolerance test (OGTT) | |  |  |  |  |  |
| Fasting Bloodwork and clotting time | |  |  |  |  |  |
| Olanzapine administration (on day 0, 1, and 2 of each visit) | |  |  |  |  |  |
| Pancreatic Euglycemic Clamp (on day 2 of each visit) (includes blood values of OLA levels, free fatty acids, triglycerides, insulin, c-peptide, glucagon, GLP-1 and catecholamines) | |  |  |  |  |  |
| 3-Day Dietary History | |  |  |  |  |  |
| SIMPAQ or IPAQ | |  |  |  |  |  |
| BAS | |  |  |  |  |  |
| FCQ | |  |  |  |  |  |
| VAS | |  |  |  |  |  |
| Urine Sample (drug screen + HCG)  Drug screen on week 2-11 is as deemed necessary by PI | |  |  |  |  |  |

* Can be combined depending on participant’s preferences and availability

** The medication (olanzapine/placebo) pick up visit will occur on Day 0 of Visits 1-3 or up to 3 days prior to Day 0. Participants will be given the option of picking up the medication blister pack, or having the medication dropped off at their place of residence.

- - 1. **MRI and cognitive testing arm**

| **Assessments** | **Week** | **0*** | **1*** | **2**** | **5**** | **8**** | **11**** |
| --- | --- | --- | --- | --- | --- | --- | --- |
|  | **Visit** | **Screening Visit 1** | **Screening Visit 2** | **1** | **2** | **3** | **4** |
| Informed Consent/Assent | |  |  |  |  |  |  |
| Demographics form | |  |  |  |  |  |  |
| Eligibility/Termination Checklist | |  |  |  |  |  |  |
| MINI | |  |  |  |  |  |  |
| Edinburgh Handedness Questionnaire | |  |  |  |  |  |  |
| Anthropometric Measures (weight, height, waist circumference) | |  |  |  |  |  |  |
| Oral glucose tolerance test (OGTT) | |  |  |  |  |  |  |
| Fasting Bloodwork (pre and post MRI) | |  |  |  |  |  |  |
| Medication Pick Up/Drop Off | |  |  |  |  |  |  |
| Olanzapine administration (on day 0, and 1 of each visit) | |  |  |  |  |  |  |
| MRI scanning (Day 2 of each visit) | |  |  |  |  |  |  |
| Cognitive testing post-MRI (Day 2 of each visit) | |  |  |  |  |  |  |
| Stanford Sleepiness scale | |  |  |  |  |  |  |
| Post procedure Bloodwork | |  |  |  |  |  |  |
| 3-Day Dietary History | |  |  |  |  |  |  |
| IPAQ or SIMPAQ | |  |  |  |  |  |  |
| BAS | |  |  |  |  |  |  |
| FCQ | |  |  |  |  |  |  |
| VAS | |  |  |  |  |  |  |
| Urine Sample (drug screen + HCG)  Drug screen on week 2-11 is as deemed necessary by PI | |  |  |  |  |  |  |
| Profile of Mood Scale | |  |  |  |  |  |  |
| Subjective Wellbeing on Neuroleptic Treatment scale | |  |  |  |  |  |  |

* Can be combined depending on participant’s preferences and availability

** The medication (olanzapine/placebo) pick up visit will occur on Day 0 of Visits 1-4, or up to 3 days prior to Day 0. Participants will be given the option of picking up the medication blister pack, or having the medication dropped off at their place of residence.

Our protocol allows for leeway in the timeframe between visits to accommodate the schedules of participants. Additionally, for participants whose participation has been interrupted, such as due to research restrictions, the time window between visits can be up to **6 months apart.** The following screening measures will need to be repeated for participants that return 12 weeks (3 months) or more since their last visit: MINI, medical history, fasting screening bloodwork.

**Participants**

Healthy non-obese volunteers (age 17-45) will be recruited through approved postings at our academic centres for the metabolic arm. This is a proof-of-concept study, based largely on work by S. Dash, examining males(31). Central insulin has sex-dependent effects in relation to appetite/food palatability(32). Sex-dependency, to our knowledge, not yet been assessed in relation to glucose metabolism while the cognitive effects on insulin have been demonstrated in both sexes (33). We will examine both sexes and investigate the effect of sex on the outcomes of interest.

Participants will be enrolled to reach complete data sets of n=27 and n=32 in the metabolic and cognitive arms of the study respectively. Therefore, participants who drop out or who are withdrawn will be replaced.

**Selection Criteria**

Inclusion Criteria – All participants

Participants of any race or ethnicity meeting all criteria listed below will be included in the study:

# Ages 17-45

Exclusion Criteria – All Participants

1. History of psychiatric illness (the Mini International Neuropsychiatric Interview (MINI) will be used to screen for psychiatric illness in the present or past Participants will be excluded for moderate to severe substance use.
2. Left handedness (only for the cognitive and MRI arm)
3. Pre-diabetes or diabetes (fasting glucose ≥6.0mmol/L or use of anti-diabetic drug);
4. Evidence of impaired glucose tolerance on screening OGTT;
5. Family history of diabetes (first degree relative such as a parent or sibling);
6. Use of weight reducing agents or other medications based on the discretion of the PI;
7. History of liver disease or AST> 2 times upper limit of normal;
8. History of kidney disease;
9. Major medical or surgical event within the last 6 months
10. Any condition that interferes with safe acquisition of MRI data such as metal implants, pacemakers, cochlear implants, claustrophobia, etc. (only for the cognitive and MRI component)
11. Pregnancy and/or breastfeeding

**Termination of the Study**

Reasons for withdrawing individual participants from the study may include one or more of the following:

1. Major protocol violation
2. Participant lost to follow-up
3. Withdrawal of consent
4. Participant pregnant or breast-feeding

Any participant may be discontinued from the study at the discretion of the investigators if this is deemed to be in the best interest of the participant. The decision may be made either to protect the participant’s health and safety, or because it is part of the research plan that people who develop certain conditions may not continue to participate.

Any research information recorded for, or resulting from, participation in this research study prior to the date that the subject formally withdrew their consent will be retained and may continue to be used and disclosed by the investigators for research purposes; however, no new data will be collected.

1. **Data Analysis Plan and Power Calculation**

Power Analysis: Using data from Dr. Dash’s previous study also employing a randomized cross-over design^32^, we found that a sample of 16 subjects would be needed to detect a 20% difference between INI-PL relative to INP-PL in endogenous glucose production, with 80% power to detect an effect size Cohen’s d = 0.66. We used a conservative estimate of the correlation between period 1 and period 2 (= 0.2). To account for the three comparisons among the three groups in the metabolic arm (using Bonferroni adjustments for multiple tests) and based on an expected attrition rate of 20% and the need to keep the total number of participants as a multiple of 3 (to counterbalance the order effects of the four treatment periods), we will recruit 27 participants to the metabolic arm of the study. To account for the six comparisons among the four groups in the cognitive arm of the study, we will recruit 32 participants to this arm. As the cognitive portion of the study is exploratory, data will be used to generate future sample sizes. The fMRI data will be analyzed using SPM12. Contrasts will be generated for activation differences between task conditions. ASL and resting state fMRI data will be analyzed using appropriate tools available in SPM12 and FSL libraries. MRS data will be analyzed using LCModel to quantify glutamate content in the VOI. Data across modalities will be analyzed in a repeated measures ANOVA design to investigate the effect of treatments. These analyses will be conducted by SMA under the supervision of AGG and AV.

To address our primary hypothesis, which is the comparison within subject between treatments, Mixed Effect Models will be used. The four treatments, time, and sequences will be entered as predictors and subject will be used as clusters in a random intercept model. In addition to examining outcome measures within subjects across treatments, predictors accounting for order effects will be included in the analyses. Mixed Effect Models will be adjusted with SAS System 9.4. A similar approach is used for cognitive data, where measures of sedation will be used as covariates.

1. **Risks/Benefits**

Blood draws for most people do not cause any serious problems. However, they may cause minor discomfort, bleeding, bruising, soreness or infections at the site on rare occasions. There may be some discomfort when the intravenous line is placed in the vein and there may be some bruising and soreness afterwards. There are no known medical risks associated with iv injection of small amounts of deuterated glucose, insulin, glucagon, somatostatin and growth hormone for the duration of the clamp. All of these substances are washed out of the system after the infusion is stopped and there are no lasting effects.

There are no known lasting effects associated with MRI scanning. Participants may perceive minor discomfort due to the scanner sounds. They will be provided with earplugs to minimize discomfort. Participants will be screened thoroughly to ensure that there are no metallic objects or implants on them.

Some of the assessments, in particular, the questionnaires, may be upsetting to study participants. We will minimize all of these by having all assessments performed by well-trained research staff with clinical experience and appropriate skills to maximize the participants’ comfort and keep their distress to a minimum during each visit.

Olanzapine is approved for the treatment of psychotic illness. It can cause side effects such as fatigue/sleepiness (26%), headache (17%), dry mouth (7%), constipation (9%), and muscle side effects. Muscle side effects may occur, and include restlessness (23%), decreased movements (<5%), muscle stiffness and rigidity (<5%) or abnormal posture of neck or hand due to muscle rigidity, called dystonia (<5%). If any of these side effects occur, they will be assessed and treated promptly.

In humans, with acute administration of intranasal insulin, no adverse effects have been reported. Moreover, in more chronic studies, 8 weeks of intranasal administration of intranasal insulin at a dose of 40 IU QID (total daily dose 160 IU) did not cause any adverse effects.

In general, placebo medication does not cause any side effects. All these side effects are temporary and will disappear (if they occur) within 24 hours of the last dose. A physician will be available at all times during the study to promptly assess and treat any side effects. A staff will always be available in evenings and overnight during the period that participants are taking study placebo/drug and not in hospital. Participants will be provided with an emergency contact card. This card will have contact information listed for study staff who will be able to contact the participant within 20 minutes of a call. In the advent of an unexpected emergency, participants are also advised they can call 911.

Hypoglycemia as a result of intranasal insulin has not been found to be a concern in the literature and across healthy volunteers there have been no reports of hypoglycemia as a result of an intranasal insulin dose equal or higher to what will be used in this protocol. In the metabolic protocol, glucose is monitored as part of the euglycemic clamp procedure and euglycemia is maintained through a peripheral glucose infusion. In the cognitive protocol, we will closely monitor for signs and symptoms of hypoglycemia, including shakiness, confusion, increased heart rate, lightheadedness, impaired vision, headaches, and weakness. In the event that any of these should occur, there will be a glucose metre and lancet in the testing room to measure glucose. If the level is below 2.8mmol/L, the study will be stopped and juice and/or glucose will be administered as deemed necessary by PI or PI designate.

**Potential Benefits of the Proposed Research to the Participants and Others**

Study participants will not directly benefit from participating in this study. This proof-of-principle clinical study aims to enhance our understanding of the mechanisms behind a very serious metabolic adverse effect of AP drugs, the risk of type 2 diabetes. Moreover, in an added exploratory aim, this study will investigate the potential cognitive impact of APs in the context of central insulin stimulation. We are reminded that no single AP is devoid of metabolic side effects in AP-naïve youth, and similarly that no single AP can reverse the cognitive deficits inherent to SCZ. Thus, this work has the potential to initiate new streams of work in an area of unmet need that intercepts disciplines of physiology, endocrinology and psychiatry.

1. **Data Management and Integrity**

The basic protection against risk in this study will be provided by Dr. Margaret Hahn (study PI). They will have primary responsibility for monitoring of participants during the entire time they participate in the study. The PI will meet regularly with study personnel to review accrued data, data confidentiality, and adherence to protocol design, recruitment, and participant complaints. During meetings the Study PI will also review the enrollment data, the accrual and integrity of clinical data, and any adverse event associated with the various components of the study. If a serious adverse event occurs during the study, it will be reported to REB.

All data pertaining to a participant’s involvement in this study will be coded and stored in locked offices. This information will only be accessible to the research team. In unusual cases, a participant’s research records may be released in response to a court order. If the research team learns that a participant or someone with whom the participant is involved with is in serious danger or harm, an investigator will inform the appropriate agencies as per legal or regulatory requirements.

The hard data are stored in a locked filing cabinet stored in a locked office to further protect participant anonymity. Data auditing, entry and quality control will be carried out at regularly. Regularly scheduled, and as needed, communications between the study team and the Study PI will clarify any inconsistencies and ambiguities in the data.

CAMH investigators will retain a participant identification code list if they need to contact participants after the study. This list will contain the complete name, identification number, address and phone number of all participants and will be held confidentially at the investigators site after completion of the study for 25 years, in accordance with Health Canada requirements.

As part of the Research Services Quality Assurance Program, this study will be regularly monitored and/or audited by a member of the Quality Assurance Team. Research records may be reviewed during which confidentiality will be maintained as per CAMH policies and extent permitted by law. As part of continuing review of the research, study records may be assessed on behalf of the Research Ethics Board.

1. **Recruitment**

Healthy volunteers will be recruited from REB-approved postings in the community , social media/online posts (e.g., University, Facebook Pages, Kijiji), and Slaight Centre at the Centre for Addiction and Mental Health (CAMH). Participants can also be recruited through the CAMH research registry for healthy volunteers, and Slaight Centre for Youth in Transition’s centralized recruitment process. Participants may be pre-screened with the REB approved “pre-screen form” that is implemented across all Slaight studies. Brief presentations can be made at university campuses/in online courses.

Participation in the study is voluntary. Participants will be informed that they have the option of terminating their participation at any time, without consequence and that no new data will be collected on them. Any existing data will be anonymized.

1. **Study Management and Materials**

CAMH Investigators will retain a participant identification code list if they need to contact participants after the study. This list will contain the complete name, identification number, address and phone number of all participants and will be held confidentially at the investigators site after completion of the study.

Study data will be entered in a secure database. An eCRF/CRF will be completed for each participant enrolled in the study. A participant screening log, noting reasons for screen failure, where applicable, will be maintained for all participants. The investigator will document the obtained informed consent and record medical and psychiatric history, medications, and efficacy data in the eCRF/CRF. Clinical scales and neuropsychological assessments will be considered source documents and will be incorporated into the eCRF/CRF in a confidential manner.

Study data will be entered in a secure database (REDCap). At point-of-entry, data values will undergo consistency edits (e.g., ID validation, range verification, duplicate detection) and personnel will be required to correct errors.

1. **Confidentiality**

There is a potential risk of breach of confidentiality that is inherent in all research protocols. Breach of confidentiality will be minimized by the staff who will maintain research data (identified only by participant code number not related to name, or date of birth) in separate charts and a dedicated password protected electronic database. A list of participant names, their ID numbers, and information about how they can be reached will be kept in a separate locked cabinet with access only to study personnel authorized by the PI. Procedures have been established, and will be followed, to minimize the risk of breach of confidentiality. Procedures to maintain confidentially include: (1) formal training sessions for all research staff emphasizing the importance of confidentiality; (2) specific procedures developed to protect participants’ confidentiality, and (3) formal mechanisms limiting access to information that can link data to individual participants. All information obtained from participants will be kept as confidential as possible. Computer based files/data will be entered into password-secured databases and paper-based files will be stored in a secure location. These data will only be accessible to personnel involved in the study and they will abide by confidentiality regulations of the REB. The ethics committee will be granted direct access to the study participants’ original medical records for verification of clinical trial procedures and/or data, without violating the confidentiality of the participants, to the extent permitted by the law and regulations.

Research data gathered as part of this study may be shared and provided to other investigators affiliated with the Schizophrenia Programme, or the Slaight Family Centre for Youth in Transition (SFCYT) at CAMH for the purpose of data sharing. If participants are enrolled in multiple studies, their research data may be shared across studies to reduce participant burden and avoid duplication of procedures. Only investigators/research team collaborating with Dr.Hahn, affiliated with the Schizophrenia Programme or SFCYT Centre will have access with Dr.Hahn’s permission to secured files and/or research data and will be well-informed regarding the protection of participants’ rights to confidentiality.

Participants will not be identified by name in any publication of research results. Results will be published as group data without the use of characteristics that would identify individual participants.

**10. Assessment of Safety**

All adverse events will be recorded in the Adverse Event Log. Adverse events include any unfavorable change in a study participant’s physical or psychological wellbeing during the study period, which may or may not be the result of participation in the study. Adverse events will be reported to the CAMH REB if all of the following REB-defined criteria are met for an **Unanticipated Problem**: 1) The event is unexpected (relative to the product monograph, research protocol, and consent forms), 2) The event is related or possibly related to participation in the research, as judged by the PI, 3) The event suggests that the research places the participants or others at a greater risk of harm than was previously known or recognized. Required reporting will occur within 48 hours of the PI becoming aware of the event. (Refer to CAMH SOP REB404).

Any **Serious Unexpected Adverse Drug Reactions** will be reported to Health Canada if all of the Health Canada-defined criteria are met: 1) The medical occurrence is serious (results in death, is life-threatening, requires hospitalization or prolongation of existing hospitalization, or is a congenital anomaly/birth defect), 2) The event is unexpected (nature or severity not consistent with information in the relevant source documents such as product monograph), and 3) It is judged by the reporting health care professional as a having a reasonable suspected causal relationship to the medicinal product. Where the event is neither fatal nor life threatening, required reporting will occur within 15 days of awareness of the information. Where it is fatal or life-threatening, it will be reported within 7 days. (Refer to Health Canada guidelines for Safety Reporting Post No-Objection Letter Section 2.8.4 and CAMH SOP HSR205).

**11. Financial Compensation**

The participant will receive compensation upon completion of each study visit (payment is made at each visit and cannot be paid in advance). The compensation schedule is mentioned in the tables below:

Metabolic arm:

| Sl. No | Visit | Compensation | Total | Description |
| --- | --- | --- | --- | --- |
| 1 | Screening | $10 + TTC tokens | $10 | Determination of eligibility for the study |
| 2 | Post screening | $30 + TTC tokens | $40 | Overnight fast and Oral glucose tolerance test |
| 3 | Medication Pick Up/Drop Off Visits (for visits 1 to 4) | TTC tokens | 2 TTC tokens | Tokens will be provided if participant opts to pick up medications |
| 4 | Visit 1 | $ 200 + TTC token + taxi drop | $240 | Olanzapine and insulin (or placebo dosing), overnight stay, pancreatic clamp |
| 5 | Visit 2 | $200 + TTC token + taxi drop | $440 | Olanzapine and insulin (or placebo dosing), overnight stay, pancreatic clamp |
| 6 | Visit 3 | $200 + TTC token + taxi drop | $640 | Olanzapine and insulin (or placebo dosing), overnight stay, pancreatic clamp |
| 6 | Study end | $200 | $840 | Gratuity for completing the study |

Cognitive arm:

| Sl. No | Visit | Compensation | Total | Description |
| --- | --- | --- | --- | --- |
| 1 | Screening | $10 + TTC tokens | $10 | Determination of eligibility for the study |
| 2 | Post screening | $30 + TTC tokens | $40 | Overnight fast and Oral glucose tolerance test |
| 3 | Medication Pick Up/Drop Off Visits (for visits 1 to 4) | TTC tokens | 2 TTC tokens | Tokens will be provided if participant opts to pick up medications |
| 4 | Visit 1 | $150 + TTC token + taxi drop | $190 | Olanzapine and insulin (or placebo dosing), overnight stay, cognitive tests, MRI scan |
| 5 | Visit 2 | $150 + TTC token + taxi drop | $340 | Olanzapine and insulin (or placebo dosing), overnight stay, cognitive tests, MRI scan |
| 6 | Visit 3 | $150 + TTC token + taxi drop | $490 | Olanzapine and insulin (or placebo dosing), overnight stay, cognitive tests, MRI scan |
| 7 | Visit 4 | $150 + TTC token + taxi drop | $640 | Olanzapine and insulin (or placebo dosing), overnight stay, cognitive tests, MRI scan |
| 7 | Study end | $200 | $840 | Gratuity for completing the study |

**References**

1. Olfson M, Blanco C, Liu L, Moreno C, Laje G. National trends in the outpatient treatment of children and adolescents with antipsychotic drugs. Arch Gen Psychiatry. 2006;63:679-685.

2. Pringsheim T, Lam D, Patten SB. The pharmacoepidemiology of antipsychotic medications for Canadian children and adolescents: 2005-2009. J Child Adolesc Psychopharmacol. 2011;21:537-543.

3. Hennekens CH, Hennekens AR, Hollar D, Casey DE. Schizophrenia and increased risks of cardiovascular disease. Am Heart J. 2005;150:1115-1121.

4. Alvarez-Jimenez M, Gonzalez-Blanch C, Crespo-Facorro B, Hetrick S, Rodriguez-Sanchez JM, Perez-Iglesias R, Vazquez-Barquero JL. Antipsychotic-induced weight gain in chronic and first-episode psychotic disorders: a systematic critical reappraisal. CNS Drugs. 2008;22:547-562.

5. Correll CU, Manu P, Olshanskiy V, Napolitano B, Kane JM, Malhotra AK. Cardiometabolic risk of second-generation antipsychotic medications during first-time use in children and adolescents. JAMA. 2009;302:1765-1773.

6. Zipursky RB, Gu H, Green AI, Perkins DO, Tohen MF, McEvoy JP, Strakowski SM, Sharma T, Kahn RS, Gur RE, Tollefson GD, Lieberman JA. Course and predictors of weight gain in people with first-episode psychosis treated with olanzapine or haloperidol. Br J Psychiatry. 2005;187:537-543.

7. Henderson DC, Cagliero E, Gray C, Nasrallah RA, Hayden DL, Schoenfeld DA, Goff DC. Clozapine, diabetes mellitus, weight gain, and lipid abnormalities: A five-year naturalistic study. Am J Psychiatry. 2000;157:975-981.

8. Oriot P, Feys JL, Mertens de Wilmars S, Misson A, Ayache L, Fagnart O, Gruson D, Luts A, Jamart J, Hermans MP, Buysschaert M. Insulin sensitivity, adjusted beta-cell function and adiponectinaemia among lean drug-naive schizophrenic patients treated with atypical antipsychotic drugs: a nine-month prospective study. Diabetes Metab. 2008;34:490-496.

9. Correll CU, Robinson DG, Schooler NR, Brunette MF, Mueser KT, Rosenheck RA, Marcy P, Addington J, Estroff SE, Robinson J, Penn DL, Azrin S, Goldstein A, Severe J, Heinssen R, Kane JM. Cardiometabolic risk in patients with first-episode schizophrenia spectrum disorders: baseline results from the RAISE-ETP study. JAMA Psychiatry. 2014;71:1350-1363.

10. Galling B, Roldan A, Nielsen RE, Nielsen J, Gerhard T, Carbon M, Stubbs B, Vancampfort D, De Hert M, Olfson M, Kahl KG, Martin A, Guo JJ, Lane HY, Sung FC, Liao CH, Arango C, Correll CU. Type 2 Diabetes Mellitus in Youth Exposed to Antipsychotics: A Systematic Review and Meta-analysis. JAMA Psychiatry. 2016;73:247-259.

11. Werner FM, Covenas R. Safety of antipsychotic drugs: focus on therapeutic and adverse effects. Expert Opin Drug Saf. 2014;13:1031-1042.

12. Vrbova K, Kamaradova D, Latalova K, Ociskova M, Prasko J, Mainerova B, Cinculova A, Kubinek R, Tichackova A. Self-stigma and adherence to medication in patients with psychotic disorders--cross-sectional study. Neuro Endocrinol Lett. 2014;35:645-652.

13. Brown S, Birtwistle J, Roe L, Thompson C. The unhealthy lifestyle of people with schizophrenia. Psychol Med. 1999;29:697-701.

14. Perry BI, McIntosh G, Weich S, Singh S, Rees K. The association between first-episode psychosis and abnormal glycaemic control: systematic review and meta-analysis. Lancet Psychiatry. 2016.

15. Chintoh AF, Mann SW, Lam L, Giacca A, Fletcher P, Nobrega J, Remington G. Insulin resistance and secretion in vivo: effects of different antipsychotics in an animal model. Schizophr Res. 2009;108:127-133.

16. Houseknecht KL, Robertson AS, Zavadoski W, Gibbs EM, Johnson DE, Rollema H. Acute effects of atypical antipsychotics on whole-body insulin resistance in rats: implications for adverse metabolic effects. Neuropsychopharmacology. 2007;32:289-297.

17. Smith GC, Chaussade C, Vickers M, Jensen J, Shepherd PR. Atypical antipsychotic drugs induce derangements in glucose homeostasis by acutely increasing glucagon secretion and hepatic glucose output in the rat. Diabetologia. 2008;51:2309-2317.

18. Martins PJ, Haas M, Obici S. Central nervous system delivery of the antipsychotic olanzapine induces hepatic insulin resistance. Diabetes. 2010;59:2418-2425.

19. Hahn MK, Chintoh A, Remington G, Teo C, Mann S, Arenovich T, Fletcher P, Lam L, Nobrega J, Guenette M, Cohn T, Giacca A. Effects of intracerebroventricular (ICV) olanzapine on insulin sensitivity and secretion in vivo: an animal model. Eur Neuropsychopharmacol. 2014;24:448-458.

20. Ikegami M, Ikeda H, Ohashi T, Ohsawa M, Ishikawa Y, Kai M, Kamei A, Kamei J. Olanzapine increases hepatic glucose production through the activation of hypothalamic adenosine 5'-monophosphate-activated protein kinase. Diabetes Obes Metab. 2013;15:1128-1135.

21. Klingerman CM, Stipanovic ME, Hajnal A, Lynch CJ. Acute Metabolic Effects of Olanzapine Depend on Dose and Injection Site. Dose Response. 2015;13:1559325815618915.

22. Sacher J, Mossaheb N, Spindelegger C, Klein N, Geiss-Granadia T, Sauermann R, Lackner E, Joukhadar C, Muller M, Kasper S. Effects of olanzapine and ziprasidone on glucose tolerance in healthy volunteers. Neuropsychopharmacology. 2008;33:1633-1641.

23. Vidarsdottir S, de Leeuw van Weenen JE, Frolich M, Roelfsema F, Romijn JA, Pijl H. Effects of olanzapine and haloperidol on the metabolic status of healthy men. J Clin Endocrinol Metab. 2010;95:118-125.

24. Teff KL, Rickels MR, Grudziak J, Fuller C, Nguyen HL, Rickels K. Antipsychotic-induced insulin resistance and postprandial hormonal dysregulation independent of weight gain or psychiatric disease. Diabetes. 2013;62:3232-3240.

25. Hahn MK, Wolever TM, Arenovich T, Teo C, Giacca A, Powell V, Clarke L, Fletcher P, Cohn T, McIntyre RS, Gomes S, Chintoh A, Remington GJ. Acute Effects of Single-Dose Olanzapine on Metabolic, Endocrine, and Inflammatory Markers in Healthy Controls. J Clin Psychopharmacol. 2013.

26. Albaugh VL, Singareddy R, Mauger D, Lynch CJ. A double blind, placebo-controlled, randomized crossover study of the acute metabolic effects of olanzapine in healthy volunteers. PLoS One. 2011;6:e22662.

27. Girgis RR, Javitch JA, Lieberman JA. Antipsychotic drug mechanisms: links between therapeutic effects, metabolic side effects and the insulin signaling pathway. Mol Psychiatry. 2008;13:918-929.

28. Obici S, Zhang BB, Karkanias G, Rossetti L. Hypothalamic insulin signaling is required for inhibition of glucose production. Nat Med. 2002;8:1376-1382.

29. Filippi BM, Yang CS, Tang C, Lam TK. Insulin activates Erk1/2 signaling in the dorsal vagal complex to inhibit glucose production. Cell Metab. 2012;16:500-510.

30. Consoli A, Nurjhan N, Reilly JJ, Jr., Bier DM, Gerich JE. Mechanism of increased gluconeogenesis in noninsulin-dependent diabetes mellitus. Role of alterations in systemic, hepatic, and muscle lactate and alanine metabolism. J Clin Invest. 1990;86:2038-2045.

31. Dash S, Xiao C, Morgantini C, Koulajian K, Lewis GF. Intranasal insulin suppresses endogenous glucose production in humans compared with placebo in the presence of similar venous insulin concentrations. Diabetes. 2015;64:766-774.

32. Kullmann S, Heni M, Hallschmid M, Fritsche A, Preissl H, Haring HU. Brain Insulin Resistance at the Crossroads of Metabolic and Cognitive Disorders in Humans. Physiol Rev. 2016;96:1169-1209.

33. Novak V, Milberg W, Hao Y, Munshi M, Novak P, Galica A, Manor B, Roberson P, Craft S, Abduljalil A. Enhancement of vasoreactivity and cognition by intranasal insulin in type 2 diabetes. Diabetes Care. 2014;37:751-759.

34. Heinrichs RW, Zakzanis KK. Neurocognitive deficit in schizophrenia: a quantitative review of the evidence. Neuropsychology. 1998;12:426-445.

35. Li J, Li X, Liu E, Copeland P, Freudenreich O, Goff DC, Henderson DC, Song X, Fan X. No effect of adjunctive, repeated dose intranasal insulin treatment on body metabolism in patients with schizophrenia. Schizophr Res. 2013;146:40-45.

36. Fan X, Copeland PM, Liu EY, Chiang E, Freudenreich O, Goff DC, Henderson DC. No effect of single-dose intranasal insulin treatment on verbal memory and sustained attention in patients with schizophrenia. J Clin Psychopharmacol. 2011;31:231-234.

37. Fan X, Liu E, Freudenreich O, Copeland P, Hayden D, Ghebremichael M, Cohen B, Ongur D, Goff DC, Henderson DC. No effect of adjunctive, repeated-dose intranasal insulin treatment on psychopathology and cognition in patients with schizophrenia. J Clin Psychopharmacol. 2013;33:226-230.

38. Chintoh AF, Mann SW, Lam L, Lam C, Cohn TA, Fletcher PJ, Nobrega JN, Giacca A, Remington G. Insulin resistance and decreased glucose-stimulated insulin secretion after acute olanzapine administration. J Clin Psychopharmacol. 2008;28:494-499.

39. Kodl CT, Seaquist ER. Cognitive dysfunction and diabetes mellitus. Endocr Rev. 2008;29:494-511.

40. Sellbom KS, Gunstad J. Cognitive function and decline in obesity. J Alzheimers Dis. 2012;30 Suppl 2:S89-95.

41. Nielsen RE, Levander S, Kjaersdam Telleus G, Jensen SO, Ostergaard Christensen T, Leucht S. Second-generation antipsychotic effect on cognition in patients with schizophrenia--a meta-analysis of randomized clinical trials. Acta Psychiatr Scand. 2015;131:185-196.

42. Born J, Lange T, Kern W, McGregor GP, Bickel U, Fehm HL. Sniffing neuropeptides: a transnasal approach to the human brain. Nat Neurosci. 2002;5:514-516.

43. Benedict RHBS, David; Groninger, Lowell; Dobraski, Melissa; Shpritz, Barnett. Revision of the Brief Visuospatial Memory Test: Studies of normal performance, reliability, and validity. . Psychological Assessment. 1996;8:145-153.

44. Lorne T. Yeudall JRR, William O. Stefanyk Normative data stratified by age and sex for 12 neuropsychological tests. Journal of Clinical Psychology. 1986;42:918-946.

45. Hoddes E, Zarcone V, Smythe H, Phillips R, Dement WC. Quantification of sleepiness: a new approach. Psychophysiology. 1973;10:431-436.

46. McLeod DR, Griffiths RR, Bigelow GE, Yingling J. An automated version of the digit symbol substitution test (DSST). Behavior Research Methods & Instrumentation. 1982;14:463-466.

47. File SE, Bond AJ. Impaired performance and sedation after a single dose of lorazepam. Psychopharmacology (Berl). 1979;66:309-313.
