# Supplementary material for: Investigating the effects of antipsychotics on brain insulin action: Study protocol for a multi-modality magnetic resonance imaging (MRI) study in healthy controls
Source: PLoS One. 2022 Nov 28;17(11):e0277211. doi: 10.1371/journal.pone.0277211 (PMC9704670; doi:10.1371/journal.pone.0277211)
Supplement: S3 File — (DOCX) [file pone.0277211.s003.docx]

**Effect of antipsychotics on central insulin action in relation to glucose metabolism and cognition in healthy volunteers**

**Informed Consent Form (Cognitive/MRI arm) –Participant Consent**

**Principal Investigators:** Dr. Margaret Hahn MD, PhD, FRCPC

Centre for Addiction and Mental Health

**Co-Principal Investigator:** Dr. Satya Dash MD, PhD, FRCPC, MRCP (UK)

**Co-Investigators:** Dr. Sri Mahavir Agarwal MBBS, MD

Dr. Adria Giacca, MD

Dr. Ariel Graff-Guerrero MD, PhD, FRCPC

Dr. Daniel Mueller MD, PhD, FRCPC

Dr. Gary Remington MD, PhD, FRCPC

Dr. Aristotle Voineskos MD, PhD, FRCPC

Dr. Valerie Taylor MD, PhD, FRCPC

**Source of funding:** Canadian Institute of Health Research (CIHR)

**Introduction and Purpose**

Insulin is a hormone in the body that controls sugar levels in part by lowering the amount of glucose produced by the liver. After eating a meal, insulin levels go up in both the blood and the brain. Insulin in the brain has also been shown to affect the way the brain works and processes information (also known as “cognition”). Antipsychotic medications may change the way brain works and processes information as well. We do not know how these changes happen and whether insulin in the brain has anything to do with this. The reason you are being asked to take part in this research study is to help find out whether insulin action in the brain has a role in bringing about these cognition related effects of antipsychotic medication. 32 participants will be included within this study group.

# Participation

1. You will be involved in this study for approximately 11-12 weeks, divided into a total of six research visits.
2. The first two visits are to determine your eligibility for the study. The next four visits will occur 2-6 weeks apart.
3. Your participation in this study will be recorded in your health record at this hospital and a copy of your consent will be added to your health record.
4. On the first visit, you will be taken through the consent form, questionnaires, a urine drug and pregnancy screen, and body measurements. This visit will take about 90 minutes.
5. In preparation for the second visit, we will ask you to fast (no food or drinks except water after 8PM). When you arrive in the morning for the second visit, we will collect a blood sample. The blood tests will monitor your general health with measurement of liver enzymes, thyroid, blood sugar, insulin, haemoglobin (blood count), inflammatory markers, and cholesterol. We will then ask you to do a glucose drink test (called a glucose tolerance test). The glucose tolerance test is used to see how your body metabolizes sugar. You will be given a sugar solution to drink. Blood will be drawn 2 hours later to measure your blood sugar. This test will make sure that you do not have prediabetes or diabetes. Only if the oral glucose tolerance test is normal will you be enrolled in the full study. A total of 6-8 teaspoons of blood will be drawn during this visit. We will also train you on the memory and attention tests that we are going to perform as a part of the study. This visit will last about 150 minutes.
6. If you are a female of child bearing age, you will have to have a urine pregnancy test. If it is positive, you will be notified and will not be asked to participate in the study because the effect of the study medication, olanzapine, is not fully known in fetuses. Furthermore, if you are breastfeeding, you also cannot participate in the study as olanzapine passes through to breast milk. You must also use an acceptable method for contraception while enrolled in the study which includes:

- Agree to abstain from sex for the duration of the trial or
- A barrier method of a diaphragm with spermicide and/or Latex condom or
- An oral contraceptive agent, implantable contraceptive or an injectable contraceptive for at least one month prior to entering the study and will continue its use throughout the study or
- An intrauterine device, or
- Partner has had a vasectomy at least 3 months prior to study start

1. If you become pregnant, or believe there is a chance you may be pregnant during the study, you must notify one of the study staff immediately.
2. You will be participating voluntarily and can withdraw from this study at any time, and this will not influence any current or future treatment at CAMH.
3. If you are eligible to take part you will be invited back for 4 further visits (2-6 weeks apart) which will involve the following:
4. On day zero, you will come to pick up your medication, and you will take a dose of olanzapine or placebo (contains no active medication) at night at your home.
5. On day one, you will come in during the evening for an overnight stay at CAMH or at a nearby hotel (cost is covered by the study). We will give you dinner that evening at approximately 5pm, which you may eat at any time up until 9:00pm, and then you cannot drink any fluid (except for water) or eat food until after the study is finished the next afternoon.. You will be asked to complete some questionnaires about your feelings of hunger, eating habits and physical activity. You will take another dose of olanzapine (or placebo) before bed.
6. On day two, we will perform fasting bloodwork.
7. After the fasting blood draw is taken, you will be given either insulin or placebo spray through the nose. Bloodwork will be repeated after the MRI and memory and attention tasks that are described below. In total, about 4-5 teaspoons of blood will be drawn during this visit and you will be poked with a needle on two occasions.
8. You will complete a brief questionnaire that will ask about sleepiness, and restlessness during the day and receive training on the memory and attention tests that we are going to perform in the MRI.
9. You will then take part in an MRI scan where your brain will be imaged. You will also complete different attention and memory tasks in the scanner. Overall, the MRI procedure will take 45-60 minutes.

- Magnetic resonance imaging (MRI) is a technology that uses strong magnetic fields (“magnetic”) and radio frequency fields (“resonance”) to produce detailed pictures of soft tissues in the body.
- Because MRI uses strong magnetic fields, we need to make sure you do not have certain metal objects in their body or with them when you enter the MRI room. You will be asked to change into hospital pants and gown when you arrive at the MRI facility. Your clothes and all personal items (e.g., watches, jewelry, wallet, cell phone) will be stored in a secure locker. The MR technologist will talk with you before the scanning session to answer any questions, and to make sure it is safe for you to go into the MRI.
- The MRI machine looks like a big doughnut, and you will lie down on a bed with both head and shoulders in the tunnel made by the “doughnut hole”. We will put some pillows around your head to keep it from moving and then ask you to stay very still while we scan your brain to get the pictures. You will be asked to try to remain as still as possible during the scans. Movements will not be dangerous in any way, but will blur the picture. For each MRI session, you will need to hold still in the machine for about 60 minutes. The MR technologist will be able to observe you at all times. You will be able to contact the MR technologist at any time during the scan session for any reason.
- You will hear moderately loud knocking or beeping sounds when the MRI machine is scanning. You will be given ear protection to wear in the scanner. Different types of scans will make different types of sounds, which is normal for MRI. The technologist will talk to you before each scan starts. There will be a mixture of short and some longer scans (up to 10 minutes each).
- Functional MRI measures your brain’s activity. For some of the scans we will ask you to rest and let your mind wander with your eyes closed, while some will ask you to watch some pictures and words and press a button to certain pictures and words so we can measure your brain’s activity. These tasks will test your ability to remember words and visual and spatial information. These scans will take about 8-9 minutes each and you will complete 2 of them.
- Once you are done with the MRI, you will take part in additional tasks which will measure the speed at which you process information and other related cognitive aspects. These will last about 45 minutes.
- You may undergo only the MRI session or the extra-scanner tests (approximately 30 min) or both, depending on feasibility and mutual and MRI slot availability.

1. After the above procedures are finished around 1:30 pm, you will receive a meal and you may go home.
2. These measures/procedures are completed 4 times, 2-4 weeks apart, throughout the study.

# Are there any risks involved?

This study has risks. Some of these risks we know about. There is also a possibility of risks that we do not know about and have not been seen in study subjects to date. Some can be managed. Please call the study doctor if you have any side effects even if you do not think it has anything to do with this study. The risks we know of are:

1. Blood draws for most people do not cause any serious problems. However, they may cause minor discomfort, bleeding, bruising, soreness or infections at the site on rare occasions. If you experience discomfort, please tell the study investigators immediately.
2. During questionnaires, you may experience some psychological discomfort while speaking to your symptoms and experiences. You are free to stop the assessments at any time.
3. Olanzapine is approved for the treatment of psychotic illness. You may not have side effects but you should know that side effects are possible. These side effects include: fatigue/sleepiness (26%), headache (17%), dry mouth (7%), constipation (9%), and muscle side effects. Muscle side effects may occur, and include restlessness (23%), decreased movements (<5%), muscle stiffness and rigidity (<5%) or abnormal posture of neck or hand due to muscle rigidity, called dystonia (<5%). Should you experience any of these side effects, you will be assessed and treated promptly.
4. In general, placebo medication does not cause any side effects. All these side effects are temporary and will disappear (if they occur) within 24 hours of the last dose. A physician will be available at all times during the study to promptly assess and treat any side-effects you may experience.
5. It is possible that intranasal insulin can impact blood glucose levels. This will be closely monitored throughout the study. If you begin to develop signs and symptoms of low glucose, such as shakiness, confusion, increased heart rate, lightheadedness, impaired vision, headaches, and weakness, we will conduct a glucose finger prick test and will treat as necessary with glucose.
6. Intranasal insulin may cause nasal irritation which resolves when discontinued. Participants may also experience mild discomfort with administration of the spray.
7. Because olanzapine can cause you to be sleepy, we ask that you do not drive before or after the study visit. We will provide you with transportation.
8. MRI scans:
9. **Metal Objects.** Before you can participate in an MRI study, we need to make sure it is safe for you to do so. Because certain metal objects may lead to injuries during the MRI procedure, we will ask you to answer questions about any metal implants or objects you might have in their body and the location of any tattoos. If you have any metal implants or objects that are not safe for the MRI at CAMH, you will not be allowed to be scanned. Some objects that are not safe for MRI include cardiac pacemakers, metal fragments in the eye, aneurysm clips in your brain. If there is a strong chance you may have metal fragments in your eyes, you will need to provide an x-ray report of your eyes before you can be scanned. The research study staff and the MR technologist will work together to make sure you will be safe in the scanner.
10. **Long-term risks.** Based on the use of MRI in medicine for over 20 years, most experts believe there are no long-term negative health effects caused by the magnetic field strength used in this study. This MRI study does not involve any form of ionizing radiation or injections.
11. **Other risks.** Some people may feel uncomfortable lying still in the confined space of the MRI scanner, tingling sensations are felt by some people during certain scans or you may feel dizzy for a few minutes at the end of the MRI study. These are infrequent, but expected sensations. It is important you understand that you will be able to contact the technologist at any time during the scan.
12. **Unexpected findings.** The possibility of unexpected or incidental findings carries with it some risks. Research scans are not designed to be used for diagnosis. In the unlikely event an atypical finding is seen on your MRI scan, we may ask a radiologist or other qualified health professional to look at the scan. By signing this consent form, you agree to allow us to release the scan for review of any unexpected findings. Your identity will not be revealed. If the qualified professional recommends further tests to determine the nature and significance of any incidental findings on your MRI scan, we will contact you to help you arrange medical follow-up.

7. If you are admitted overnight in a hotel, you will be provided with an emergency contact card with the names and contact information of study staff. If in the unlikely event of any sort of an emergency, you can call the number of a staff listed on the card and they will be able to reach you within 20 minutes of your call. In the advent of an emergency, you can also call 911. You will also be able to reach a staff member on the nights you are at home taking the medication/placebo.

# Are there any benefits involved?

This study will not directly benefit you. It will help benefit others by helping scientists understand early how medications like olanzapine impact metabolic health and the role of insulin in these processes.

# What if I am injured during/in this study?

# If you suffer an injury from participation in this study, medical care will be provided to you in the same manner as you would ordinarily obtain any other medical treatment.  In no way does signing this consent form waive you or your legal rights nor release the study doctor(s), sponsors or involved institutions from their legal and professional responsibilities. If you require treatment for any injuries or illness related to your participation in the study, or if you suffer side effects while on a study drug, you should contact the study doctor immediately.

# Compensation

| Sl. No | Visit | Compensation | Total | Description |
| --- | --- | --- | --- | --- |
| 1 | Screening | $10 + 2 TTC tokens | $10 | Determination of eligibility for the study |
| 2 | Post screening | $30 + 2 TTC tokens | $40 | Overnight fast and Oral glucose tolerance test |
| 3 | Visit 1 | $150 + 3 TTC tokens + taxi drop | $190 | Olanzapine and insulin (or placebo dosing), overnight stay, cognitive tests, MRI scan |
| 4 | Visit 2 | $150 + 3 TTC tokens + taxi drop | $340 | Olanzapine and insulin (or placebo dosing), overnight stay, cognitive tests, MRI scan |
| 5 | Visit 3 | $150 + 3 TTC tokens + taxi drop | $490 | Olanzapine and insulin (or placebo dosing), overnight stay, cognitive tests, MRI scan |
| 6 | Visit 4 | $150 + 3 TTC tokens + taxi drop | $640 | Olanzapine and insulin (or placebo dosing), overnight stay, cognitive tests, MRI scan |
| 7 | Study end | $200 | $840 | Gratuity for completing the study |

# Confidentiality

All information that identifies you will be kept confidential and stored and locked in a secure place that only the study personnel will have access to. In addition, electronic files will be stored on a secure hospital or institutional network and will be password protected. The investigators will dispose of your paper and computer-based records after the research obligations for the study have been met. Confidentiality will be respected and no information that discloses your identity will be released or published without consent, unless required by law.

There are three exceptions to our confidentiality policy. In any of the following situations, we are obligated by law to contact authorities: 1) if there is a serious possibility that you may harm yourself or others, 2) if you have been involved in any form of child abuse or neglect, 3) if you have been the victim of abuse by a healthcare worker.

A description of this clinical trial will be available at http://www.ClinicalTrials.gov. This website will not include information that can identify you. At most, the website will include a summary of the results. You can search this website at any time.

*As part of the Research Services Quality Assurance Program, this study may be monitored and/or audited by a member of the Quality Assurance Team. Your research records may be reviewed during which confidentiality will be maintained as per CAMH policies and extent permitted by law.*

*As a part of continuing review of the research, your study records may be assessed on behalf of the Research Ethics Board. A person from the research ethics team may contact you (if your contact information is available) to ask you questions about the research study and your consent to participate. The person assessing your file or contacting you must maintain your confidentiality to the extent permitted by law*.

Research data gathered as part of this study may be shared and provided other investigators collaborating with Dr. Hahn, affiliated with the Schizophrenia Programme, and Slaight Family Centre for Youth in Transition (SFCYT) for the purpose of data sharing. If you are enrolled in multiple studies in the Schizophrenia Programme or the SFCYT, your research data will be shared across studies to reduce participant burden and avoid duplication of procedures. Only investigators/research team affiliated with these teams will have access to secured files and / or to master lists for participant code numbers and/or research data and will be well-informed regarding the protection of participants’ rights to confidentiality.

This study is under the authority of Health Canada. Your records may therefore be assessed by the Health Canada Therapeutic Products Programme.

# Right to Refuse or Withdraw from the Study

Your participation in this study is voluntary. You may refuse to participate or may stop participation at any time without penalty. You should also understand that the Principal Investigator running this study, Dr. Margaret Hahn, may stop your participation at any time. The decision may be made either to protect your health and safety, or because it is part of the research plan that people who develop certain conditions may not continue to participate. Throughout your participation in this study, you may continue regular appointments with your original treating physician.

**New Information**

If new information becomes available that is relevant to your participation to continue in the study, you will be informed in a timely manner.

Any research information recorded for, or resulting from, your participation in this research study prior to the date that you formally withdrew your consent may continue to be used and disclosed by the investigators for the purposes described above; however, no new data will be collected.

# Offer to Answer Questions

We have used some technical terms in this form. Please feel free to ask about anything that you do not understand. Consider this research and the consent form carefully as long as you feel necessary before you make a decision.

Dr. Margaret Hahn is responsible for this study. If you have any questions, please contact Dr. Margaret Hahn at 416-535-8501 x 34368. If you have any questions about your rights as a participant in a research study, you may contact Dr. Robert Levitan, Chair, Research Ethics Board, Centre for Addiction and Mental Health, at 416 535 8501 ext. 34020.

# Consent to Participate

**My signature below indicates that:**

- I have read the above information and have been given the opportunity to ask further questions about this study.
- The study, the inconveniences, risks and benefits have been explained to me and my questions have been answered.
- I know that I can ask further questions at any stage during the study.
- I understand that I will not benefit directly from this study.
- I understand that I may withdraw from this study at any time.
- I understand that if interested, I will be asked to complete a urine sample that will be used to check for street drugs, alcohol, and other medications.
- I understand if I am female, I will have to have a pregnancy test. If it is positive, I will be notified and will not be asked to participate in the study.
- I understand that if I am breastfeeding, I cannot participate in the study.
- I also consent to have the data collected from this study entered in an anonymized manner, that is, without information that could identify me as the subject, in a research database that is shared by other investigators. The purpose is to compare with data from patients with several conditions and with those of healthy controls of the same sex and age.

I have been given a copy of this Consent Form to keep and I agree to participate in this study. By signing this consent form I am not giving up any of my legal rights.

I have had the chance to talk about this study with and my questions have been answered to my satisfaction. If I have more questions, I can contact Dr. Hahn at 416-535-8501, Ext. 34368.

Participant’s Signature Date

:

Print Participant’s Name Time

Signature of Person Obtaining Consent Date

:

Print Name of Person Obtaining Consent Time

# Future Contact

Our Centre conducts new studies on an ongoing basis. Please let us know if you would be interested in being contacted by our research staff about future studies for which you may be eligible to participate:

Yes, I agree to be contacted for future research studies.

No, I do not wish to be contacted for future research studies.

Participant’s Signature Print Participant’s Name Date
