# Supplementary material for: Investigating the effects of antipsychotics on brain insulin action: Study protocol for a multi-modality magnetic resonance imaging (MRI) study in healthy controls
Source: PLoS One. 2022 Nov 28;17(11):e0277211. doi: 10.1371/journal.pone.0277211 (PMC9704670; doi:10.1371/journal.pone.0277211)
Supplement: S4 File — (DOCX) [file pone.0277211.s004.docx]

**Supplementary File 4**

**MRI Acquisition Parameters**

- 1. Structural MRI: High-resolution T1 weighted anatomical image will be acquired using the BRAVO sequence to yield a voxel size of 0.9 × 0.9 × 0.9 mm^3^ (BRAVO, TR=6.8 ms; TE=3.0 ms; flip-angle 8°; 256x256 matrix; FOV=23 cm; slice thickness = 0.9 mm).
  2. Resting state functional MRI (rs-fMRI): A 10-minute resting-state echo-planar imaging (EPI) scan will be acquired for each participant (49 axial slices; slice thickness=3.0 mm; TR=2250 ms; TE=30 ms; matrix 64x64; FOV 20 cm). Participants are instructed to rest quietly with their eyes open and to remain awake during the scan. BOLD-[Blood Oxygen Level Dependent] signaling will be used to measure functional brain activity at rest.
  3. Task-based fMRI paradigm testing visuospatial cognitive skills: fMRI data will be acquired during the performance of the spatial n-back paradigm, a visuospatial working memory task [27] (49 axial slices; slice thickness=3.0 mm; TR=2250 ms; TE=30 ms; matrix 64x64; FOV 20 cm).
  4. Arterial spin labelling (ASL): ASL images will be acquired to measure blood perfusion within the striatum and dorsolateral prefrontal cortex (DLPFC) (TR=4612 ms ; TE=10.6 ms; FOV= 22cm; slice thickness= 4.0 mm).
  5. ^1^H-Magnetic Resonance Spectroscopy (^1^H-MRS): Single voxel spectra will be acquired for a volume of interest (VOI) placed over frontal and striatal regions of interest (TR=2000 ms; TE=35 ms; FOV=24 cm; NEX=8.0; voxel dimensions: striatum: 15mm x 25mm x 20mm; DLPFC: 15mm x 30mm x 30mm).

**Spatial n-back intrascanner cognitive task**

Each block will be run six times, starting with the 0-back and then alternating between the two conditions for a total duration of about four minutes. Before each block, an instruction slide will inform the participant about the nature of the block. The participants will have to respond to all stimuli requiring constant updating of the working memory as well as continuous response execution. Participants will be trained on the working memory task for as long as they wish prior to the experiment to achieve an overall task accuracy level of more than 70% before entering the scanner.
