# Supplementary material for: Investigating the effects of antipsychotics on brain insulin action: Study protocol for a multi-modality magnetic resonance imaging (MRI) study in healthy controls
Source: PLoS One. 2022 Nov 28;17(11):e0277211. doi: 10.1371/journal.pone.0277211 (PMC9704670; doi:10.1371/journal.pone.0277211)
Supplement: S5 File — (PDF) [file pone.0277211.s005.pdf]

camh

November 7, 2022

PLOS ONE Editorial Manager
plosone@plos.org

To Whom It May Concern:

RE: PONE-D-22-26140 - Investigating the effects of antipsychotics on brain insulin action: Study protocol for a multi-modality magnetic resonance imaging (MRI) study in healthy controls

This letter is regarding the above noted study protocol that was recently accepted for publication in PLOS ONE. In publishing this study protocol, it is understood that it will be freely available online, and any third party is permitted to access, download, copy, distribute, and use these materials in any way, even commercially, with proper attribution, as per the Creative Commons Attribution (CC BY) 4.0 license. On behalf of the of the Research Operations, Services, and Support Office at the Centre for Addiction and Mental Health (CAMH), the sponsoring institution for this study, I confirm that we can comply with this policy and give permission for the study protocol to be published with PLOS ONE.

Thank you for the opportunity to publish this work.

Regards,

Aristotle Voineskos

Aristotle Voineskos, MD, PhD, FRCP(C)
Vice President, Research & Director, Campbell Family Mental Health Research Institute, CAMH
Professor, Department of Psychiatry, University of Toronto
